# Unveiling the Role of the Lewis Acids in the Acceleration of Alder-Ene Reactions: A Molecular Electron Density Theory Study

**DOI:** 10.3390/molecules30214289

**Published:** 2025-11-04

**Authors:** Luis R. Domingo, Patricia Pérez

**Affiliations:** 1 Independent Researcher, Avd. Tirso de Molina 20, 46015 Valencia, Spain; 2 Facultad de Ciencias, Departamento de Ciencias Biológicas y Químicas, Universidad San Sebastián, Campus Ciudad Universitaria, Av. del Condor 720, Ciudad Empresarial, Huechuraba, Santiago 8580704, Chile

**Keywords:** Lewis acid catalysts, Alder-ene reactions, global electron density transfer, electrophilicity, molecular electron density theory, relative interacting atomic energies

## Abstract

The electronic effects of Lewis acids (LAs) in reducing the activation energies of Alder-ene (AE) reactions have been studied within the Molecular Electron Density Theory (MEDT). To this end, the AE reactions of 2-methylbutadiene (2MBD) with formaldehyde (CHO) in the presence of three LAs with increasing acidic character, BH_3_, BF_3_, and AlCl_3_, have been studied. Topological analysis of the electron density and the evaluation of the DFT-based reactivity indices indicate that LAs do not modify the electronic structure of the carbonyl group but markedly increase the electrophilic character of CHO. LAs not only strongly accelerate the AE reactions, but also modify the molecular mechanisms, changing them from a non-concerted *two-stage one-step* mechanism to a two-step one. Topological analyses of the electron density at the transition state structures (TSs) indicate that while the formation of the new C-C single bond has begun, the departure of the hydrogen has not yet started. A Relative Interacting Atomic Energy (RIAE) analysis of the activation energies allows the establishment of the electronic effects of LAs on the AE reactions. LAs increase the global electron density transfer (GEDT) occurring in polar AE reactions; this phenomenon markedly stabilizes the CHO framework at the TSs, decreasing the RIAE relative energies.

## 1. Introduction

**Scheme 1:**
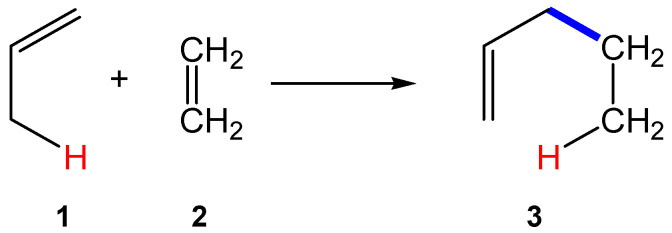
AE reaction of propene **1** with ethylene **2**.

The ene reaction [1], first proposed by Alder in 1943 [2,3], involves an alkene with an allylic hydrogen and an ethylene. This reaction is one of the simplest methods to form a C-C single bond. In the simplest Alder-ene (AE) reaction, propene **1** reacts with ethylene **2** to form a new C-C single bond accompanied by the migration of the allylic hydrogen and the propene C=C double bond, resulting in pentene **3** (see Scheme 1).

**Scheme 2:**
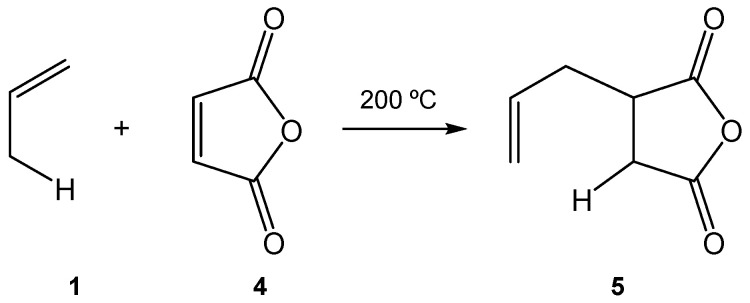
AE reaction of propene **1** with maleic anhydride **4**.

However, the high activation energy associated with this hydrocarbon AE reaction renders it impractical to perform experimentally. Therefore, to experimentally carry out an AE reaction, the ethylene must be activated. Consequently, propene **1** reacts with maleic anhydride **4** at 200 °C to produce adduct **5** (see Scheme 2) [4,5].

**Scheme 3:**
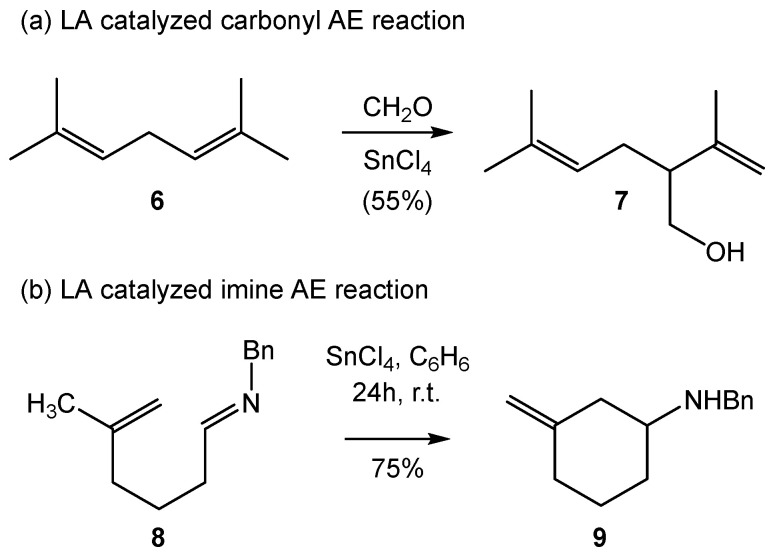
LA activated AE reactions of aldehydes and imines.

Carbonyl and imine compounds, bearing a C=O and a C=N double bond, respectively, can participate as heteroethylenes in AE reactions [6], but they require the presence of a Lewis acid (LA) to proceed under mild conditions (see Scheme 3).

**Scheme 4:**
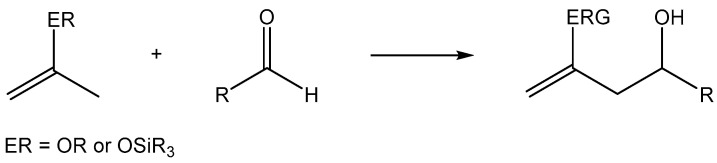
Activation of the propene component through the presence of ER groups.

On the other hand, the presence of electron-releasing (ER) groups, such as an alkoxy group, -OR, and a silyloxy group, -OSiR_3_, at the C2 carbon of propene significantly increases the reactivity of propene **1** (see Scheme 4) [7,8,9,10].

**Scheme 5:**
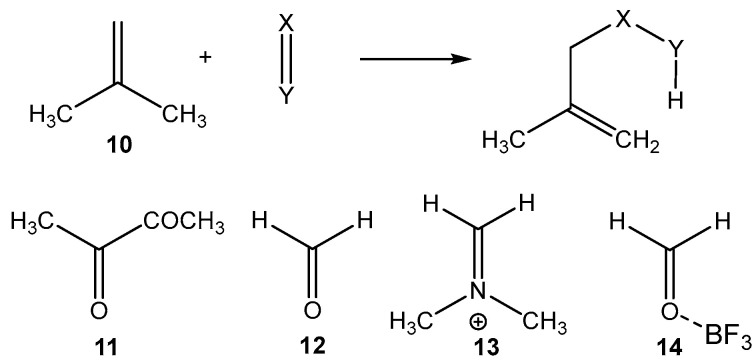
AE reactions of isobutene **10**, showing selected heteroethylene derivatives.

The AE reactions have been the focus of multiple theoretical studies [11,12,13]. In 2014, the AE reactions of isobutene **10** with twelve ethylenes and heteroethylenes of increased electrophilic character were studied at the MPWB1K/6-311G(d,p) level (see Scheme 5) [14]. The activation energies of the AE reactions ranged from 34.7 kcal·mol^−1^ for the reaction with ethylene **2** to −1.2 kcal·mol^−1^ for the BF_3_-catalyzed reaction with formaldehyde (CHO) **12**. Most of the heteroethylenes were carbonyl or carboxyl compounds such **11**, **12** and **14**.

**Figure 1:**
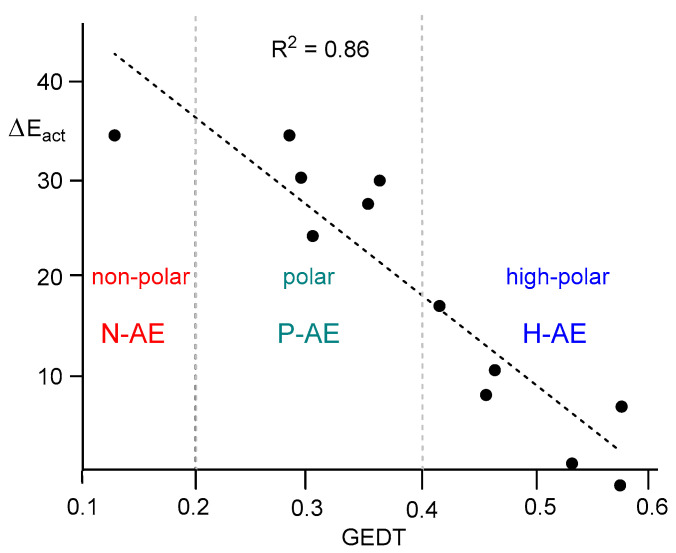
Plot of the activation energies, ΔE_act_, in kcal·mol^−1^, vs. the GEDT values, in average number of electrons, e, for the AE reactions shown in Scheme 5.

Similar to Diels-Alder (DA) reactions [15], a strong correlation between the polar character of the AE reaction, as measured by the global electron density transfer [16] (GEDT) at the transition state structures (TSs), and the computed activation energy was found (see Figure 1) [14]. This behavior allowed establishing a useful classification of AE reactions in non-polar (N-AE) reactions, characterized by very high activation energy, greater than 35 kcal·mol^−1^ and GEDT values < 0.20 e (ethylene **2**); polar (P-AE) reactions, with activation energies between 20 and 35 kcal·mol^−1^ and GEDT values of 0.20 ≤ GEDT ≤ 0.40 e (methyl acetate **11** and CHO **12**); and high-polar (H-AE) reactions, featuring activation energies below 20 kcal·mol^−1^ and GEDT values > 0.40 e (iminium cation **13** and CHO:BF_3_ complex **14**) [14]. Thus, while hydrocarbon N-AE reactions are experimentally unfeasible, LA-catalyzed heteroethylene H-AE reactions take place at room temperature.

Electron localization function [17] (ELF) topological analysis of the changes in electron density along the reaction paths allowed the characterization of the non-concerted one-step mechanism for P-AE reactions, which involves an initial nucleophilic/electrophilic two-center interaction, followed by the hydrogen transfer process occurring at the end of the reaction. This topological analysis of the bonding changes established that the mechanisms of the P-AE reactions are non-concerted, thereby discarding the proposed pericyclic mechanism [18] for AE reactions [19].

After twenty years devoted to the theoretical study of organic chemical reactivity, Domingo proposed the Molecular Electron Density Theory [20] (MEDT) in 2016 to study chemical organic reactivity. This theory states that the energy cost associated with the reorganization of the molecular electron density along a reaction path determines chemical reactivity. Consequently, analyzing the changes in electron density along the reaction path provides a new perspective to that offered by molecular orbital (MO) interaction models, such as the Frontier Molecular Orbital theory proposed in 1964 [21]. In this context, MEDT focuses on electron density–based descriptors rather than orbital interactions or energy decomposition schemes derived from MO analyses, including the activation strain model (ASM) [22]. Over the past years, MEDT has been successfully applied to investigate a wide variety of organic reactions, offering valuable insights into their underlying mechanisms.

**Scheme 6:**
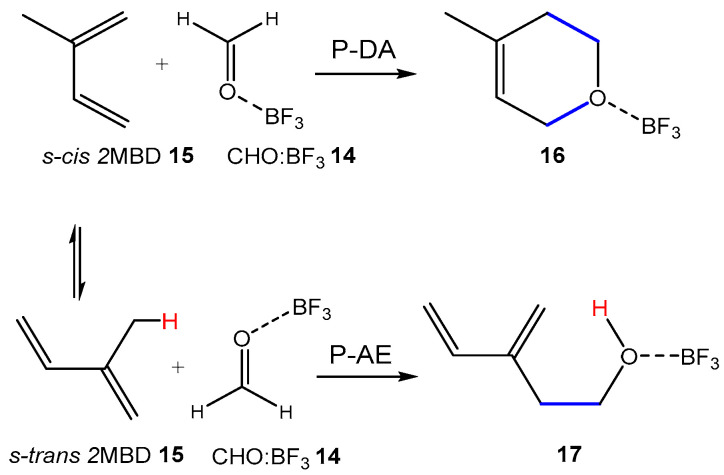
Competitive P-DA and P-AE reactions of 2MBD **15**, a diene possessing an allylic hydrogen, with CHO:BF_3_ **14**.

Due to the similarity between the mechanisms of the P-DA reactions [15], proposed in 2009, and the P-AE reactions, the competitive P-DA and P-AE reactions of 2-methyl-1,3-butadiene (2MBD, **15**), a nucleophilically activated propene with an ER vinyl group, and CHO:BF_3_ **14**, a carbonyl compound electrophilically activated by the presence of BF_3_ LA, were studied within MEDT in 2018 (see Scheme 6) [23]. While the P-DA reaction proceeds via a non-concerted one-step mechanism, the competitive H-AE reaction takes place via a two-step mechanism through the formation of a zwitterionic intermediate. Both P-DA and P-AE reactions were characterized by the initial C-C single bond formation associated with the nucleophilic attack of the C3 carbon of 2MBD **15** on the carbonyl C1carbon of CHO:BF_3_ **14**.

**Scheme 7:**
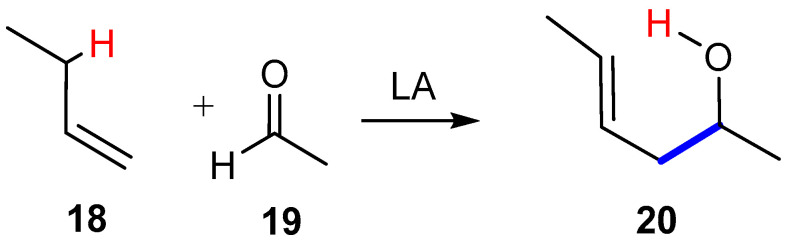
LA catalyzed AE reaction between 1-butene **18** and acetaldehyde **19**.

In 2023, Fernández et al. studied LA-catalyzed carbonyl-ene reaction between 1-butene **18** and acetaldehyde **19** in the presence of a series of LAs by using the ASM (see Scheme 7) [24]. These authors concluded that the catalytic role of the LAs on the carbonyl-ene reactions mainly involves a significant reduction in the ‘Pauli repulsion’ between the key occupied π-molecular orbitals of the reactants, rather than the widely accepted stabilization of the LUMO of acetaldehyde **19**. This proposal was similar to one previously made in LA-catalyzed DA reactions [25].

Herein, it is important to note that at the TS of the AlCl_3_-catalyzed AE reaction between 1-butene **18** and acetaldehyde **19**, the C1-C3 distance was 1.913 Å [23], indicating that the formation of the new C1-C3 bond had already begun [16]. Consequently, at this C-C distance, the concepts of Pauli repulsion and molecular orbital interactions should be interpreted carefully, as they no longer offer a physically meaningful description of the system.

Recently, an energy decomposition analysis (EDA) based on the Interacting Quantum Atoms [26] (IQA), namely the Relative Interacting Atomic Energy [27] (RIAE), was introduced within the MEDT framework. This EDA enables the analysis of electronic atomic interactions responsible for the activation energies. IQA, which is based on the AIM [28,29], allows the partitioning of the total energy calculated via Density Functional Theory [30] (DFT) into intra- and interatomic energy components. Based on the Kohn-Sham [31] (KS) approach, the total energy of a molecule is divided into kinetic and potential energy contributions associated with individual atoms and their intra- and inter- interactions.

Carbonyl derivatives are typically classified as electrophilic species that undergo a wide range of nucleophilic addition processes [32]. Nevertheless, aldehydes and ketones do not readily undergo addition reactions with neutral nucleophiles due to the moderate electrophilic character of these substrates [33]. To favor the nucleophilic additions, LA catalysts are commonly employed, as they significantly enhance the electrophilicity of the carbonyl group [27].

**Scheme 8:**
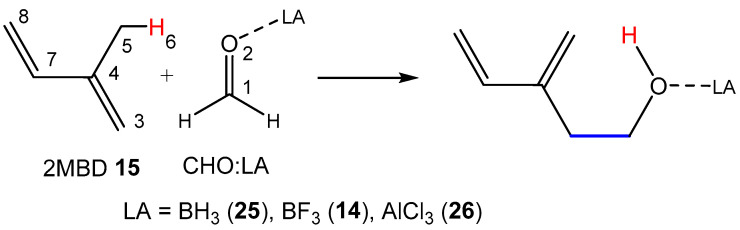
LA catalyzed AE reactions of 2MBD **15** and CHO **12**.

Due to the relevance of the LAs as catalysts in polar organic reactions involving moderate electrophilic carbonyl compounds [27], and the fact that LAs permit the AE reactions involving hetereoethylenes as carbonyl and imine compounds to take place easily even at room temperature (see Scheme 2), the AE reactions of 2MBD **15**, a nucleophilic activated propene, with CHO **12**, a carbonyl compound acting as an electrophilic heteroethylene, in the presence of three LAs of increased acidic character, BH_3_, BF_3_ and AlCl_3_, are herein studied within MEDT (see Scheme 8). An RIAE analysis of the non-catalyzed and the three selected LA-catalyzed AE reactions is carried out herein to establish the electronic effects caused by the presence of the LAs in the decrease in the activation energies of AE reactions.

## 2. Results and Discussion

This MEDT study is organized into five sections: (i) initially, the topological analysis of the ELF of the electronic structure of the reagents is conducted, along with an analysis of the chemical properties based on the DFT-based reactivity indices [34,35] in the ground state (GS) of the reagents; (ii) second, the non-catalyzed AE reaction of 2MBD **15** with CHO **12** is examined; (iii) third, the LA-catalyzed AE reactions of 2MBD **15** with CHO **12**, using three common LAs of increasing acidic character, BH_3_, BF_3_ and AlCl_3_, are explored; (iv) fourth, the effects of the LAs on the kinetics of the LA-catalyzed AE reactions are analyzed; and finally, (v) the fifth section presents an RIAE analysis of the non-catalyzed and LA-catalyzed AE reactions to establish the electronic effects caused by the presence of the LAs in reducing the activation energies of these organic reactions.

### 2.1. Study of the Electronic Structure and Chemical Properties of the Reagents in the GS

#### 2.1.1. Study of the Electronic Structure of the Reagents

**Figure 2:**
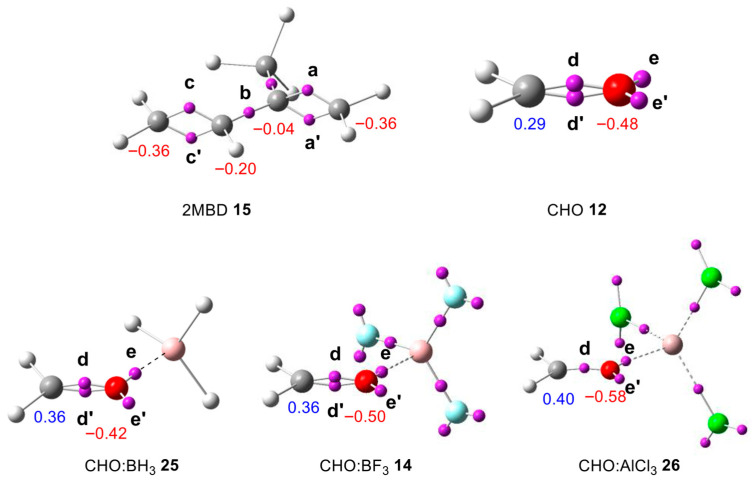
ELF basin attractor positions of 2MBD **15**, CHO **12** and CHO:LA complexes **14**, **25** and **26**, calculated at the *ω*B97X-D/6-311G(d,p) level in the gas phase. Natural atomic charges are reported as an average number of electrons, e. Negative charges are highlighted in red, and positive charges are shown in blue.

Before studying the LA-catalyzed AE reactions of 2MBD **15** with CHO **12**, the electronic structures of reagents at the GS were investigated through topological analysis of the ELF [17]. The ELF permits a quantitative characterization of the electron density distribution in a molecule [36]. The attractor positions of the ELF basins and the natural atomic charges of 2MBD **15**, CHO **12**, and three representative CHO:LA complexes **14**, **25**, and **26** are shown in Figure 2, while the populations of the more relevant valence basins are given in Table 1.

ELF topological analysis of 2MBD **15** reveals the presence of two disynaptic basins, V(C3,C4) and V’(C3,C4), integrating a total of 3.42 e, associated with a depopulated C3-C4 double bond, one V(C4,C7) disynaptic basin integrating 2.22 e, associated with a populated C4-C7 single bond, and two disynaptic basins, V(C7,C8) and V’(C7,C8), integrating a total of 3.40 e, associated with a depopulated C7-C8 double bond. The methyl group at the C5 carbon does not substantially modify the ELF of *s-trans* butadiene (see the Figure S1 in the Supplementary Material). In 2MBD **15**, the total electron density of the C=C-C=C framework is increased by 0.13 e due to the ER character of the C5 methyl group.

ELF topological analysis of CHO **12** shows the presence of two disynaptic basins, V(C1,O2) and V’(C1,O2), integrating a total of 2.46 e, associated with a highly depopulated C1-O2 double bond, and two monosynaptic basins, V(O2) and V’(O2), integrating 5.08 e, corresponding to the non-bonding electron density of the O2 oxygen atom. This electron density distribution results from the noticeable polarization of the electron density of the C1-O2 bonding region toward the strongly electronegative O2 oxygen atom.

Coordination of the three selected LAs; BH_3_, BF_3_, and AlCl_3_, to the carbonyl O2 oxygen of CHO causes slight changes in the electron density of the C-O bonding region of the three LA complexes **14**, **25**, and **26**. Thus, while the C-O bonding region experiences variations in Δe = −0.04 (**25**, BH_3_), 0.00 (**14**, BF_3_), and 0.08 (**26**, AlCl_3_), the non-bonding O2 electron density varies by Δe = 0.12 (**25**, BH_3_), 0.03 (**14**, BF_3_), and 0.27 (**26**, AlCl_3_). Note that while LA complexes **14** and **25** display two V(C1,O2) and V’(C1,O2) disynaptic basins, LA complex **26** shows only one V(C1,O2) disynaptic basin due to the strong polarization of the carbonyl group upon AlCl_3_ coordination.

The natural atomic charges of the most relevant centers are presented in Figure 2. The natural population analysis [37,38] (NPA) of 2MBD **15** shows that the four carbons of the butadiene system are negatively charged as a consequence of the greater electronegativity of the carbon atom than the hydrogen atom. Thus, the C4 carbon that is bonded to three carbons has a negligible charge −0.04 e. The NPA of CHO **12** shows that, while the carbonyl C1 carbon is markedly positively charged at +0.29 e, the O2 oxygen atom is strongly negatively charged at −0.48 e. This charge distribution results from the strong electronegative character of the O2 oxygen atom.

Coordination of the LAs to the oxygen atom of CHO **12** increases the positive charge of the carbonyl C1 carbon by between +0.07 and +0.11 e and decreases the negative charge of the oxygen by 0.06 e in CHO:BH_3_ **25**, while increasing it by 0.02 and 0.10 e in CHO:BF_3_ **14** and CHO:AlCl_3_ **26**, respectively. The AlCl_3_ LA causes the strongest changes in the electron density distribution of CHO **12**. Consequently, coordination of these LAs to the carbonyl O1 oxygen of CHO **12** does not substantially modify the electronic structure of the carbonyl C=O group at GS.

#### 2.1.2. Analysis of the Chemical Properties of the Reagents

A useful method for comprehending reactivity in polar reactions is the analysis of DFT-based reactivity indices [34,35]. Subsequently, the DFT-based reactivity indices at the GS of the reagents were examined to study the chemical reactivity of the CHO and the corresponding CHO:LA complexes. The B3LYP/6-31G(d) computational level was used, as it was employed to establish electrophilicity and nucleophilicity scales [35]. Table 2 summarizes the computed B3LYP/6-31G(d) global reactivity indices, including electronic chemical potential *μ*, chemical hardness *η*, electrophilicity ω and nucleophilicity *N* indices. The *ω*B97X-D/6-311G(d,p) global reactivity indices are given in Table S1 in the Supplementary Material.

The electronic chemical potential [39] μ of 2MBD **15**, μ = −3.34 eV, is above that of CHO **12**, μ = −4.23 eV, and those of the CHO:LA complexes **14**, **25** and **26**, which range from −5.29 to −6.09 eV. This suggests that in these polar AE reactions, the GEDT [16] will occur from 2MBD **15** to CHO **12** and CHO:LA complexes **14**, **25**, and **26**, in reactions classified as forward electron density flux [40] (FEDF).

Ethylene **2** has electrophilicity [41] ω and nucleophilicity [42] *N* indices of 0.73 and 1.86 eV, respectively, being classified as a marginal electrophile and a marginal nucleophile. Consequently, ethylene **2** will have no tendency to participate in polar reactions. This behavior accounts for the very high activation energy found in the AE reaction of isobutene **10** and ethylene **2**, 34.7 kcal·mol^−1^ [14].

The electrophilicity and nucleophilicity of propene **1** and 1-butene **18**, ca. 0.60 and 2.32 eV, respectively, classify these species as marginal electrophiles and moderate nucleophiles. These low values indicate that these alkenes will effectively participate in P-AE reactions only with superelectrophilic heteroethylenes with ω > 4.0 eV. The inclusion of a second ER CH_3_ methyl group in propene **1** increases the nucleophilicity of isobutene **10** to 2.60 eV.

2MBD **15** has electrophilicity ω and nucleophilicity *N* indices of 0.99 and 2.97 eV, respectively. It is classified as a moderate electrophile and is located at the borderline of strong nucleophiles. Consequently, 2MBD **15** will participate in polar reactions with strong electrophilic species [23].

CHO **12** has electrophilicity ω and nucleophilicity *N* indices of 1.45 and 1.81 eV, respectively, and is, therefore, classified as a moderate electrophile and a marginal nucleophile. Consequently, CHO **12** will have a poor tendency to participate in polar reactions as an electrophile [33].

Coordination of the LAs to the carbonyl O2 oxygen atom of CHO **12** markedly increases the electrophilicity ω index of the corresponding CHO:LA complexes **14**, **25** and **26**, between Δω = 1.28 (**25**, BH_3_) and 2.90 (**26**, AlCl_3_) eV. Thus, while CHO:BH_3_ **25**, ω = 2.87 eV, and CHO:BF_3_ **14**, ω = 2.73 eV, are categorized as strong electrophiles, the CHO:AlCl_3_ **26**, ω = 4.35 eV, with ω > 4.00 eV, is categorized as a superelectrophile.

The inclusion of an ER CH_3_ methyl group in the carbonyl group of CHO **12** decreases the electrophilic character of MeCHO **19**, ω = 1.12 eV, and that of the corresponding MeCHO:AlCl_3_ complex **21**, ω = 3.62 eV. These behaviors permit an explanation of the lower reactivity of these species compared to CHO **12** and CHO:AlCl_3_ **26** when participating in AE reactions (see below).

### 2.2. Study of the Non-Catalyzed AE Reaction of 2MBD ***15*** with CHO ***12***

**Scheme 9:**
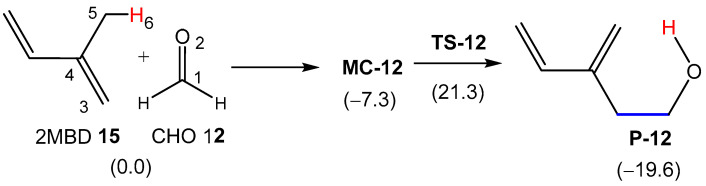
Non-catalyzed AE reaction of 2MBD **15** with CHO **12**. The *ω*B97X-D/6-311G(d,p) relative energies, in kcal·mol^−1^, are given in parentheses.

Before studying the LA-catalyzed AE reactions of 2MBD **15** with CHO **12**, the potential energy surface (PES) of the corresponding non-catalyzed AE reaction was investigated (see Scheme 9). One molecular complex (MC), **MC-12**, one TS, **TS-12**, and the en-ol product **P-12** were located and characterized along the reaction path. Consequently, this AE reaction takes place through a one-step mechanism. The *ω*B97X-D/6-311G(d,p) relative energies are given in Scheme 9, and the total energies are provided in Table S2 in the Supplementary Material.

Along the reaction path, an MC was found at the beginning of the reaction on the PES. **MC-12** is located 7.3 kcal·mol^−1^ below the separated reagents. **TS-12** is located 21.3 kcal·mol^−1^ above the separated reagent, the AE reaction being exothermic by 19.6 kcal·mol^−1^. The activation energy for this AE reaction is 13.4 kcal·mol^−1^ lower than that between isobutene **10** and ethylene **2**, due to the low polar character of the latter [14]. Despite this reduction, the activation energy remains high because of the moderate electrophilic nature of CHO **12** [33]. Note that the AE reaction of 1-butene **18** with MeCHO **19**, given in Scheme 7 [24], has an activation energy of 31.3 kcal·mol^−1^ in dichloromethane as a consequence of the low nucleophilic character of **18** and the low electrophilic character of **19** (see Table 2).

**Figure 3:**
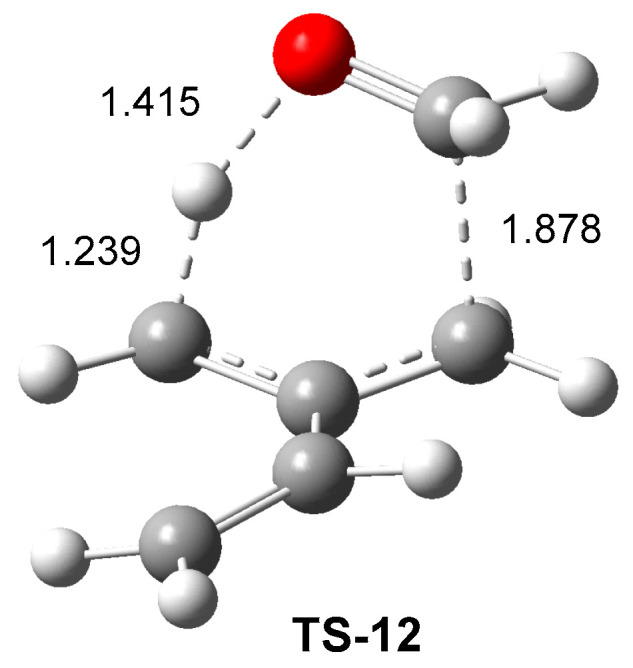
*ω*B97X-D/6-311G(d,p) optimized geometry of **TS-12**. Distances are given in Angstroms.

The optimized geometry of **TS-12** is shown in Figure 3. The distance between the C3 carbon of 2MBD **15** and the carbonyl C1 carbon of CHO **12** is 1.878 Å, while the distances from the H6 hydrogen atom to the C5 carbon and O2 oxygen are 1.239 and 1.415 Å, respectively. These geometrical parameters indicate that the formation of the new C1-C3 single bond has already begun at this TS, while the hydrogen transfer process is still delayed (see below). Note that the C-C bond formation typically begins within the range of 1.90–2.0 Å [16], while the O-H distance is shorter than the C-H one.

Analysis of the GEDT [16] at **TS-12** involved in this AE reaction allows for quantifying its polar character [14]. The sign of the GEDT computed at the TS unambiguously allows the classification of polar reactions as FEDF, with GEDT > +0.05 e, or REDF, with GEDT < −0.05 e [40]. The sign of the GEDT values indicates that the propene framework, i.e., the hydrogen donor, is positively or negatively charged, meaning that propene acts as a nucleophile or electrophile at the corresponding TS. Non-polar reactions characterized by a negligible GEDT ≤ |0.05| e are classified as null electron density flux [40] (NEDF). The GEDT at the 2MBD **15** framework of **TS-12** is 0.27 e. This high value indicates that the high polar character of this AE reaction is characterized by FEDF. Note that the GEDT at the AE reaction of isobutene **10** with ethylene **2** is 0.13 e [14].

Both, the activation energy associated with the AE reaction of 2MBD **15** with CHO **12**, and the GEDT taking place at **TS-12** classify the reaction as a P-AE reaction [14].

**Figure 4:**
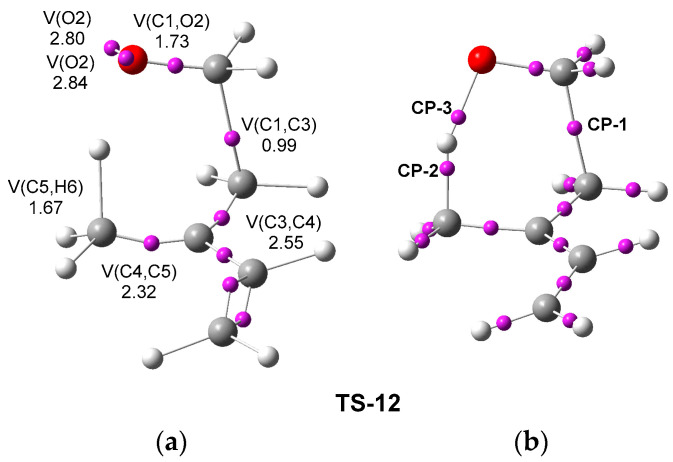
(**a**) *ω*B97X-D/6-311G(d,p) ELF basin attractor positions and populations, in average number of electrons, e, of the most relevant valence basins; and (**b**) position of the AIM (3,−1) CPs of **TS-12**.

Next, an ELF [17] and AIM [28,29] topological analysis of the electron density of **TS-12** was performed in order to understand the bonding changes taking place at this TS. The ELF basin attractor positions and the most relevant valence basin populations of **TS-12** are given in Figure 4a, while the position of the AIM (3,−1) critical points (CPs) is given in Figure 4b.

The ELF of **TS-12** shows the presence of a V(C1,C3) disynaptic basin integrating 0.99 e, indicating that the formation of the new C1-C3 single bond has already begun at this TS [16]. On the other hand, the presence of the V(C5,H6) disynaptic basin integrating 1.67 e indicates that the C5-H6 single bond has not broken.

The AIM [28,29] topological analysis allows for comprehending the type of interatomic interactions at the C1-C3, C5-H6, and O2-H6 interacting regions of **TS-12**. QTAIM parameters, namely the total electron density *ρ* and Laplacian of electron density ∇^2^*ρ* at the CPs associated with these interacting regions at the **TS-12** are listed in Table 3, while the position of the CPs is shown in Figure 4b.

The AIM of **TS-12** shows the presence of a CP-1 in the C1-C3 interacting region with an electron density ρ = 0.2765 e, and a negative Laplacian, ∇^2^*ρ*, indicating a covalent interaction. On the other hand, CP-2, with ρ = 0.1880 e and a negative Laplacian, and CP-3, with a ρ = 0.1031 e, and a positive Laplacian, indicate that the C5-H6 single bond has not been broken, and the formation of the O2-H6 single bond has not yet begun.

Consequently, both ELF and AIM topological analyses of the electron density of **TS-12** indicate that it corresponds to an asynchronous process, in which the formation of the C1-C3 single bond has already started, while the rupture of the C5-H6 single bond has not yet started.

### 2.3. Study of the LA-Catalyzed AE Reactions of 2MBD ***15*** with CHO ***12***

**Scheme 10:**
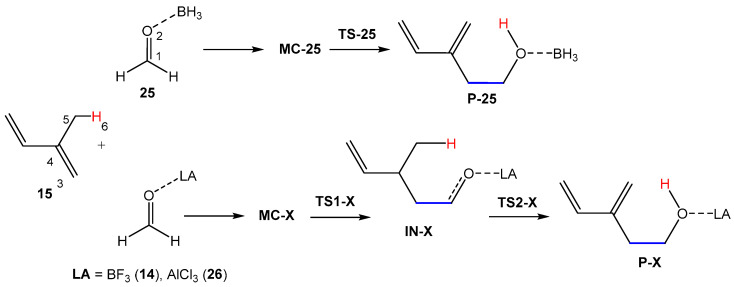
One-step and two-step mechanisms of the LA-catalyzed AE reactions of 2MBD **15** with CHO **12**.

Next, the PESs of the LA-catalyzed AE reactions of 2MBD **15** with CHO **12** involving three common LAs of increasing acidic character: BH_3_, BF_3_, and AlCl_3_, were studied. Analysis of the stationary points along the PES of these LA-catalyzed AE reactions indicated that, while the AE reaction involving the BH_3_ takes place through a one-step mechanism, the other two AE reactions involving more acidic LAs take place through a two-step mechanism (see Scheme 10). The relative energies of the TSs, intermediates, and products are given in Table 4, while the total energies are provided in Table S2 in the Supplementary Material.

Several conclusions can be obtained from the relative energies given in Table 4: (i) along the three reaction paths, one MC is found in an early stage of the reaction. These MCs are found between 10.3 (**MC-25**, BH_3_) and 13.7 (**MC-26**, AlCl_3_) kcal·mol^−1^ below the separated reagents; (ii) the three reactions are exothermic, with values ranging between 25.4 (**P-25**, BH_3_) and 30.4 (**P-26**, AlCl_3_) kcal·mol^−1^; (iii) while **TS-25** is located 3.3 kcal·mol^−1^ above the separated regents, the TSs associated with the first step of the stepwise mechanisms are located −4.3 (**TS1-14**) and −10.3 (**TS1-26**) kcal·mol^−1^ below the separated reagents; (iv) if the formation of the MCs is considered, the activation energies become positive by 7.4 (**TS1-14**) and 3.4 (**TS1-26**) kcal·mol^−1^; and finally, (v) the two intermediates **IN-X** are stabilized by only 0.0 (**IN-14**) and 0.5 (**IN-26**) kcal·mol^−1^ with respect the corresponding **TS1-X**, while the activation energies associated with the proton transfer are less than 1.3 kcal·mol^−1^. Thus, in the gas phase, the PESs around **IN-14** and **IN-26** are very flat.

The activation energy for the BH_3_-catalyzed AE reaction via **TS-25** is 18.0 kcal·mol^−1^ lower than that for the non-catalyzed reaction via **TS-12**, indicating the strong effect of the BH_3_ catalyst. In the other two LA-catalyzed reactions, the relative energies of the TSs involved in the nucleophilic attack of 2MBD **15** on the carbonyl C1 of CHO:BF_3_ **14** and CHO:AlCl_3_ **26** are lowered by 7.6 (**TS1-14**) and 13.0 (**TS1-26**) kcal·mol^−1^ compared to **TS-25**. These highlights show that the nature of the LA catalyst influences the reaction rate of these AE reactions.

**Figure 5:**
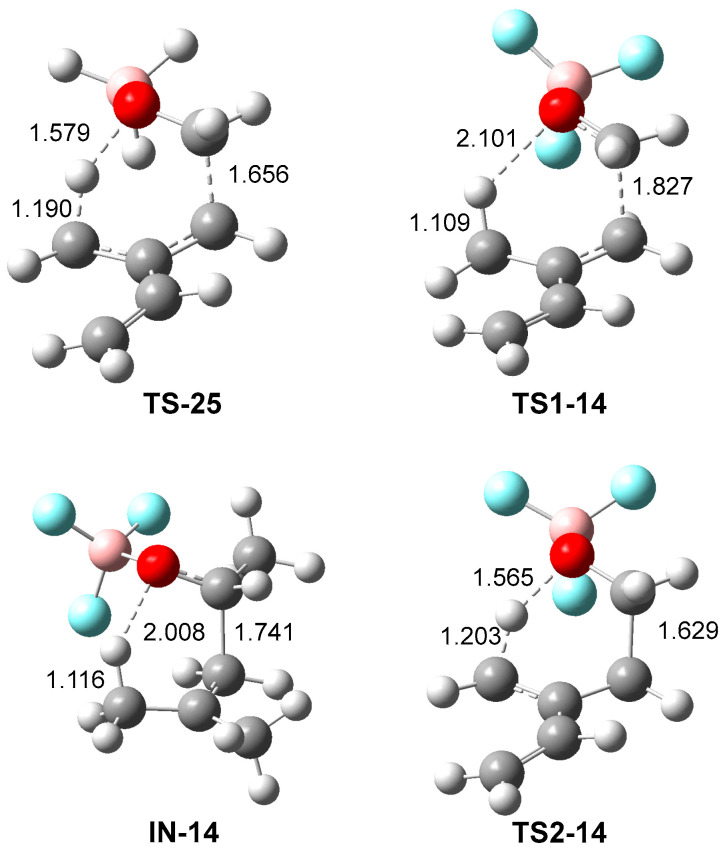
*ω*B97X-D/6-311G(d,p) optimized geometry of TSs and intermediate involved in the BH_3_ and BF_3_ LA-catalyzed AE reactions of 2MBD **15** with CHO **12**. Distances are given in Angstroms.

The geometries of the TSs and intermediates involved in the BH_3_ and BF_3_ LA-catalyzed AE reactions of 2MBD **15** with CHO **12** are shown in Figure 5, while the main geometrical parameters of the TSs and intermediates involved in the three LA-catalyzed AE reactions are listed in Table 5.

The C1-C3, C5-H6, and O2-H6 distances at the TSs associated with the nucleophilic attack of 2MBD **15** on the C1 carbon of CHO in the BH_3_ and BF_3_ LA-catalyzed AE reactions are found within the ranges 1.66–1.83 Å, 1.11–1.19 Å, and 1.58–2.10 Å, respectively. Consequently, the O2-H6 distance at **TS-25**, 1.579 Å, is less than the other parameters, which are very similar among the three TSs. The short C1-C3 distance at all three TSs, less than 1.827 Å, indicates that, similar to **TS-12**, the formation of the C1-C3 single bond has already begun. On the other hand, the C5-H6 and O2-H6 distances at **TS1-14** and **TS1-26** indicate that the transfer of hydrogen H6 to oxygen O2 has not yet begun.

The C1-C3, C5-H6, and O2-H6 distances at the two intermediates **IN-14** and **IN-25** are 1.68 and 1.74 Å, 1.12 Å for both, and 2.00 and 2.01 Å, respectively. These distances are close to those at **TS1-14** and **TS1-26** as a consequence of the highly advanced nature of the TSs and the very flat PES in these two-step reactions.

The C1-C3, C5-H6, and O2-H6 distances at **TS2-14** and **TS2-26** are 1.63 and 1.61 Å, 1.20 and 1.23 Å, and 1.57 and 1.52 Å, respectively. The shorter C5-H6 distances, approximately 1.21 Å, compared to the O2-H6 distance, roughly 1.54 Å, suggest that the proton transfer processes occur much later at the two TSs. The C5-H6 and O2-H6 distances at the two TSs are closer to those in **TS-12**.

The C5-H6 distances at **TS1-14** and **TS1-26**, and **IN-14** and **IN-26**, lesser than 1.12 Å, are only slightly longer than the other two allylic C5-H6 bonds, 1.09 Å. These observations, together with the similarly large O2-H6 distances at both TSs and intermediates, between 2.00 and 2.10 Å, suggest that the O2-H6 interactions in four species should be considered as a strong intramolecular O2^…^H6 hydrogen bond interaction rather than a hydrogen transfer process, as at **TS-12**.

Finally, a comparison of the geometrical parameters of **TS-25** and **TS2-14**, as shown in Figure 5, reveals a strong similarity between them, suggesting that the former is mainly associated with the hydrogen transfer process.

The GEDTs at **TS-25**, **TS1-14**, and **TS1-26** are 0.41, 0.48, and 0.63 e, respectively (see Table 5). These high values indicate the strong polar character of these LA-catalyzed AE reactions characterized by FEDF. These values are markedly higher than those of the non-catalyzed AE reaction via **TS-12**, 0.27 e, in agreement with the greater electrophilic character of the CHO:LA complexes (see Table 2). The maximum GEDT value is obtained at intermediates **IN-14** and **IN-26**, as GEDT depends on the electrophilic/nucleophilic character of the two interacting species, and their separation, i.e., the shorter the separation, the higher the GEDT.

The low activation energies associated with the LA-catalyzed AE reactions of CHO **12**, which are lower than 20 kcal·mol^−1^, and the high GEDT values (>0.40 e) found at the TSs, allow these reactions to be classified as H-AE reactions, which can take place at room temperature [14].

The activation energy of the AE reaction of 1-butene **18** with MeCHO:AlCl_3_ **21** is 6.1 kcal·mol^−1^ [24]. This activation energy is 16.2 kcal·mol^−1^ higher than that the relative energy for the AE reaction of 2MBD **15** with CHO:AlCl_3_ **26**, −10.1 kcal·mol^−1^ (see Table S2 in Supplementary Material). This energy difference is a consequence of the moderate nucleophilic character of 1-butene **18**, and the lower electrophilic character of MeCHO:AlCl_3_ **21** than CHO:AlCl_3_ **26** (see Table 2). This finding reveals the importance of analyzing the DFT-based reactivity indices of the reagents to predict the polar character of an AE reaction, and consequently its feasibility (see Figure 1).

**Figure 6:**
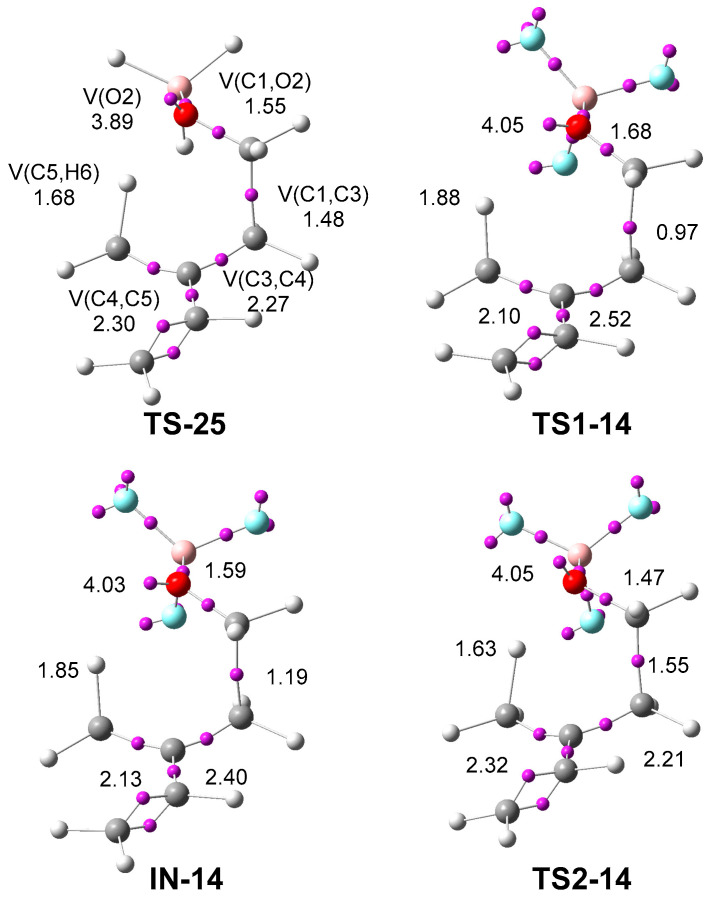
*ω*B97X-D/6-311G(d,p) ELF basin attractor positions and populations, expressed as the average number of electrons, e, of the most relevant valence basins of **TS-25**, **TS1-14**, **IN-14,** and **TS2-14**. The populations of the selected valence basins of **TS1-14**, **IN-14**, and **TS2-14** correspond with the valence basins of **TS-25**.

Next, an ELF topological analysis of the TSs and intermediates involved in the BH_3_ and BF_3_ LA-catalyzed AE reactions of 2MBD **15** with CHO **12** was performed. The ELF basin attractor positions and the most relevant valence basin populations of **TS-25**, **TS1-14**, **IN-14,** and **TS2-14** are shown in Figure 6.

The four species show the same number and types of valence basins as those at **TS-12**, but they present different populations. ELF of **TS-12** and **TS-25** shows a great similarity (see Figure 4 and Figure 6). **TS-25** shows a new V(C1,C3) disynaptic basin integrating 1.48 e. This population is higher than that at **TS-12**, indicating the more advanced character of the former. The presence of this disynaptic basin shows that the formation of the new C1-C3 single bond has already begun. On the other hand, the presence of the V(C5,H6) disynaptic basin integrating 1.68 e indicates that the C5-H6 single bond has not broken.

The ELF topological analysis of **TS1-14**, **IN-14**, and **TS2-14** shows a great similarity between these species. Only some changes in the population are observed because of the evolution of the bonding changes along the reaction path. These behaviors agree with the closer relative energies and geometrical parameters of these species, in agreement with a very flat PES. Thus, the population of the V(C1,C3) disynaptic basins at the three species is 0.97, 1.19, and 1.55 e, respectively, showing the advancement in the formation of the C1-C3 single bond. Note that the population of the V(C1,C3) disynaptic basins at **P-14** is 1.99 e (see Figure S2 in the Supplementary Material). Meanwhile, although the population of the V(C5,H6) disynaptic basin remains around 1.9 e at **TS1-26** and **IN-26**, which is closer to the other two V(C5,H) disynaptic basins, at 2.0 e, it slightly decreases to 1.63 e at **TS2-26**. In the three species, the population of the V(O2) monosynaptic basin remains at 4.05 e. These behaviors, along with the analysis of the geometric parameters, suggest that both **TS1-14** and **TS1-26** are solely related to the nucleophilic attack of 2MBD **15** on the C1 carbon of CHO:LA complexes **14** and **26**. This attack is favored by the strong hydrogen bond formation between the carbonyl O2 oxygen and the H6 hydrogen present on the C5 methyl group, which will be transferred after passing **TS2-26**.

Finally, the populations of the selected disynaptic basins of **TS-25** are very close to those of **TS2-14**, indicating the similarity between the electronic structure of the two TSs. These results indicate that the more favorable **TS1-14** is more delayed than **TS-25**.

### 2.4. Study of the Effects of the LAs on the Kinetics of the LA-Catalyzed AE Reactions

Next, the effects of the LAs on the kinetics of the LA-catalyzed AE reactions of 2MBD **15** with the CHO:LA complexes **14**, **25**, and **26** were examined through a comparative analysis of the relative reaction rate constants (RRRCs), k_r,_ computed using the Eyring-Polanyi equation [43]. To this end, all gas phase stationary points involved in the AE reactions of 2MBD **15** with CHO **12**, and with the CHO:LA complexes **14**, **25**, and **26** were first optimized in dioxane. Subsequently, the thermodynamic data were computed by frequency calculations performed in this solvent at 25 °C. The AE reaction of 2MBD **15** with the CHO **12** was used as the reference reaction.

Table 6 summarizes the calculated thermodynamic parameters, including the activation Gibbs free energies, while Table 7 contains the RRRCs, k_r_, values relative to the AE reaction of 2MBD **15** with the CHO **12**. The thermodynamic data of the stationary points are listed in Table S3 in the Supplementary Material, and the main geometrical parameters of TSs and intermediates optimized in dioxane are shown in Table S4 in the Supplementary Material.

Solvent effects stabilize the TSs and intermediates by 0.4 to 4.1 kcal·mol^−1^ due to their polar character but destabilize MCs and products by 0.9–1.7 kcal·mol^−1^. Despite these variations, solvent effects do not substantially modify the gas phase PESs. In addition, solvent effects do not produce remarkable changes in the gas-phase optimized geometries (see Table S4 in the Supplementary Material).

Some valuable conclusions can be drawn from the thermodynamic parameters provided in Table 6: (i) including thermal correction to the electronic energies only slightly modifies the relative enthalpies of TSs by between 0.0 and 2.2 kcal·mol^−1^, and those of the intermediates by 2.8 kcal·mol^−1^. As a result, the relative enthalpies follow the same trend as the relative electronic energies; (ii) including the entropies and temperature in the enthalpies increases the relative Gibbs free energies between 10.0 and 16.7 kcal·mol^−1^, due to the unfavorable activation entropies associated with these bimolecular processes. (iii) although the formation of the MCs is exothermic ranging from −4.6 to −9.8 kcal·mol^−1^, they are endergonic by between 1.0 and 3.4 kcal·mol^−1^; (iv) the relative Gibbs free energies of the TSs involved in nucleophilic attack of 2MBD **15** on the C1 carbon of CHO in these LA-catalyzed AE reactions are endergonic by between 4.5 and 15.7 kcal·mol^−1^, showing the three LA-catalyzed AE reactions positive activation Gibbs free energies; and finally, (v) for the AE reaction of 2MBD **15** with CHO:BF_3_, **25** the second step becomes the rate-determining step (RDS) of this stepwise reaction, which is **TS2-14**, 0.8 kcal·mol^−1^ above **TS1-14** in Gibbs free energy.

**Figure 7:**
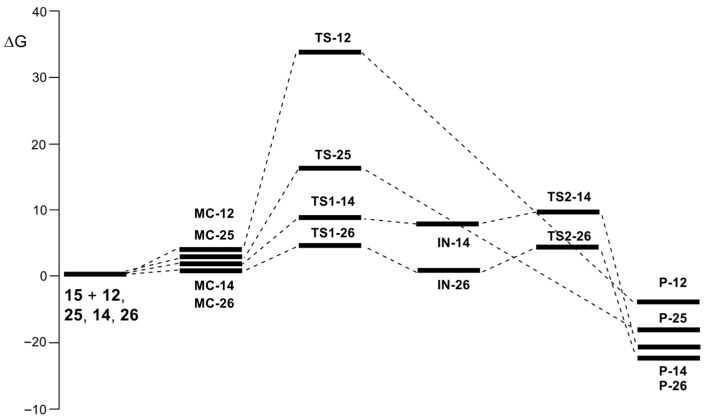
Gibbs free energy profiles, computed at 25 °C in dioxane for the AE reactions of 2MBD **15** with CHO **12**, and with the CHO:LA complexes **14**, **25** and **26**. Relative Gibbs free energies are given in kcal·mol^−1^.

The Gibbs free energy profiles for the AE reactions of 2MBD **15** with CHO **12** and with the CHO:LA complexes **14**, **25**, and **26** are shown in Figure 7. As observed, LAs have a significant impact on both the kinetic and thermodynamic aspects of these AE reactions. The activation Gibbs free energies of the LA-catalyzed AE reactions are significantly lowered by 18.3 to 29.5 kcal·mol^−1^ compared to the non-catalyzed process, while the exergonic nature of these AE reactions is enhanced by 4.8 to 9.2 kcal·mol^−1^. As can be seen, the formation of the four MC is endergonic, while all activation Gibbs free energies are positive. The most noticeable change in the analysis of the Gibbs free energy profiles is that for the LA-catalyzed AE reaction between 2MBD **15** and CHO:BF_3_ **14**, the second step involving proton transfer becomes the RDS of this stepwise AE reaction (see Figure 7).

Finally, the RRRCs, k_r,_ constants for the LA-catalyzed AE reactions of 2MBD **1** with the CHO:LA complexes **14**, **25,** and **26**, relative to the non-catalyzed AE reaction of 2MBD **15** with CHO **12**, were computed using the Eyring-Polanyi equation [43]. The corresponding values are given in Table 7. As can be seen, the coordination of an LA to the oxygen atom of CHO **12** causes a significant acceleration of the corresponding AE reactions, resulting in a k_r_ between 2.5 × 10^13^ and 4.5 × 10^21^ times higher compared to the non-catalyzed process. These high k_r_ values indicate the high efficiency of the LAs as catalysts of AE reactions involving carbonyl compounds. Note that the rate constants from the Eyring–Polanyi equation do not include possible compensation effects [44]; thus, the acceleration factors in Table 7 are qualitative, illustrating the enhancement of the reaction rate with the presence of the LAs.

**Figure 8:**
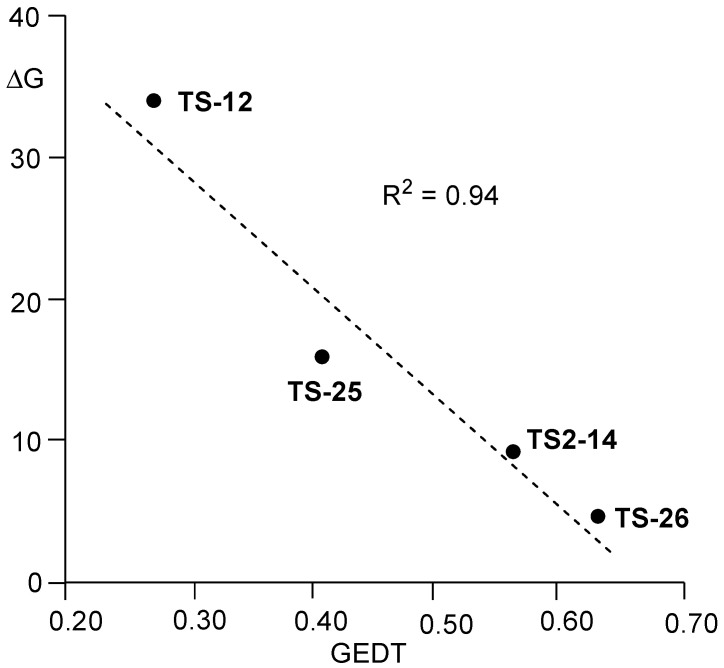
Plot of the activation Gibbs free energies, in kcal·mol^−1^, associated with the RDS of the non-catalyzed AE reactions of 2MBD **15** with the CHO **12**, and the LA-catalyzed AE reactions of 2MBD **15** with the CHO:LA complexes **14**, **25**, and **26** versus the GEDT at the corresponding TSs, as the average number of electrons, e, at the corresponding TS.

As shown in Figure 8, a strong linear correlation between the activation Gibbs free energies associated with the RDS of the non-catalyzed and LA-catalyzed AE reactions and GEDT determined at the corresponding TS is found, R^2^ = 0.94. Consequently, as in the LA-catalyzed DA reactions [27], a strong correlation between the polar character of the reaction measured by the GEDT computed at the corresponding TSs, and the feasibility of the AE reaction can be established (see below).

### 2.5. RIAE Analysis of the Electronic Effects of LAs on the AE Reactions of 2MBD ***15*** with CHO ***12***

To gain deeper insight into the electronic factors governing the activation barriers, an EDA based on the RIAE approach [27] was performed to evaluate the electronic effects of the LAs on the activation energies of the AE reactions of 2MBD **15** with CHO **12** and with the CHO:LA complexes **25**, **14**, and **26**. This analysis was applied to the regents and TSs involved in the nucleophilic/electrophilic interaction step of these AE reactions, providing insight into the energy costs associated with electron density reorganization on going from the reagents to the TSs, on which MEDT is founded [20]. The theoretical background of the RIAE method is outlined in the Supplementary Material. As a reference, the AE reaction of 2MBD **15** with ethylene **2** via **TS-2** was also considered. The RIAE study was carried out at the M06-2X/6-311G(d,p) computational level in the gas phase, as required by the IQA [26] calculations. The gas-phase ξ$E_{intra}^{X}$ intra-atomic, ξ$E_{inter}^{X}$ interatomic and ξ$E_{total}^{X}$ total energies for the 2MBD and the CHO frameworks of the four TSs are presented in Table 8. The sum of the ξ$E_{total}^{X}$ values of both frameworks, denoted as ξ$E_{total}^{2MBD+CHO}$, represents the RIAE relative energy.

**RIAE** analysis of **TS-2**, corresponding to the AE reaction of 2MBD **15** with ethylene **2**, reveals that the main contributor to the RIAE relative energy is the destabilization of the 2MBD framework, ξ$E_{total}^{2MBD}$ = 27.30 kcal·mol^−1^. Interestingly, the ethylene framework is only destabilized by 1.88 kcal·mol^−1^. Analysis of the unfavorable contribution to the ξ$E_{total}^{2MBD}$ total energies shows that the unfavorable intra-atomic energies of the 2MBD framework, ξ$E_{intra}^{2MBD}=$ 44.24 kcal·mol^−1^, are the primary factors responsible for the height of the high RIAE relative energy, 29.10 kcal·mol^−1^.

RIAE analysis of **TS-12**, corresponding to the non-catalyzed AE reaction of 2MBD **15** with CHO **12**, shows a reduction in the RIAE relative energy by 8.40 kcal·mol^−1^ with respect to that of the AE reaction of 2MBD **15** with ethylene **2**. Even though the 2MBD framework is more strongly destabilized, ξ$E_{total}^{2MBD}$ = 48.88 kcal·mol^−1^, the CHO framework is stabilized by −28.18 kcal·mol^−1^. Note that while ethylene **2** is a marginal electrophile, CHO **12** is a moderate electrophile, thus favoring the GEDT from 2MBD to the CHO framework. Analysis of the factors responsible for the stabilization of ξ$E_{total}^{CHO}$ indicates that the strong intra-atomic stabilization of the CHO framework, ξ$E_{intra}^{CHO}$ = −109.50 kcal·mol^−1^, is the main factor responsible for the reduction in the RIAE relative energy.

RIAE analysis of the LA-catalyzed AE reactions helps understand the electronic effects of the LAs in lowering the activation energies of these LA-catalyzed AE reactions. The RIAE relative energies of the three LA-catalyzed AE reactions decrease as the acidic character of the LA increases, with values of 1.54 (**TS-25**), −5.02 (**TS1-14**), and −10.05 (**TS1-26**) kcal·mol^−1^. In fact, the LA-catalyzed AE reactions involving BF_3_ and AlCl_3_ show negative RIAE relative energies, meaning that the TS associated with the first step is electronically more stabilized than the separated reactants.

Analysis of the factors responsible for this trend shows that while the nucleophilic 2MBD framework is destabilized by ξ$E_{total}^{2MBD}$ = 44.94 (**TS-25**), 54.53 (**TS1-14**) and 71.04 (**TS1-26**) kcal·mol^−1^, the electrophilic CHO:LA framework is strongly stabilized by ξ$E_{total}^{CHO-Al}$ = −43.40 (**TS-25**), −59.56 (**TS1-14**) and −81.09 (**TS1-26**) kcal·mol^−1^, with the increase in the acidic character of the LA. A detailed analysis of the factor responsible for these stabilizations reveals that, similar to the non-catalyzed process, the intra-atomic stabilization of the electrophilic CHO:LA framework, ξ$E_{intra}^{CHO-LA}$ = −76.27 (**TS-25**), −83.58 (**TS1-14**) and −121.99 (**TS1-26**) kcal·mol^−1^, is responsible for the decrease in the RIAE relative energies. A detailed analysis of the factor responsible for the increased stabilization of the CHO:AL framework indicates that the strong stabilization of the carbonyl C1 carbon, ξ$E_{intra}^{C1}$ = −68.12 (**TS-25**), −86.86 (**TS1-14**) and −98.98 (**TS1-26**) kcal·mol^−1^, is the main factor responsible.

**Figure 9:**
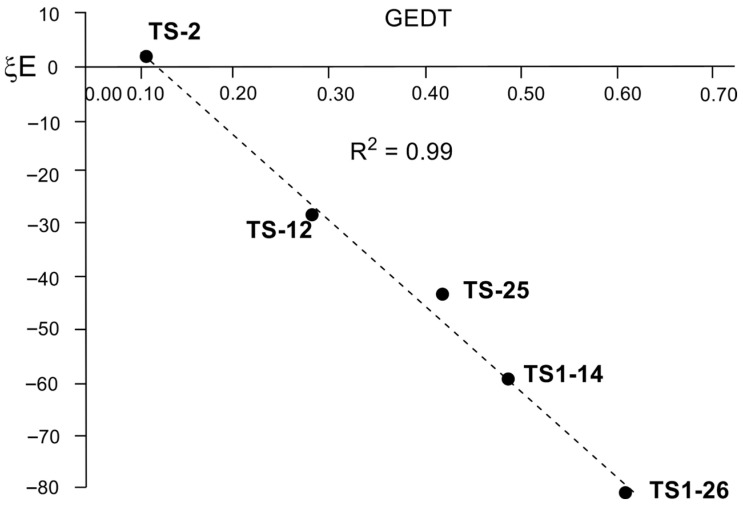
Plot of the ξ$E_{total}^{CHO}$ total energies of the CHO framework, ξE in kcal·mol^−1^, versus GEDT at the corresponding TSs, as the average number of electrons, e.

As shown in Figure 9, a strong linear correlation between the ξ$E_{total}^{CHO}$ total energies of the CHO framework and GEDT at the corresponding TSs are found, R^2^ = 0.99. As in polar DA reactions [27,45], the present RIAE analysis demonstrates that the GEDT occurring at the TSs of these polar AE reactions, primarily at the carbonyl C1 atom, is the main factor responsible for lowering the RIAE relative energies in the LA-catalyzed AE reactions. In other words, increasing the acidic character of the LA enhances the electrophilic nature of the CHO:LA complex (see Table 2), which increases the GEDT and thereby reduces the activation energies [27]. Thus, similar to the LA-catalyzed DA reaction, coordination of the LAs to the oxygen atom of carbonyl compounds increases the electrophilicity of these species, promoting the GEDT at the TS, and consequently, lowering the activation barrier energies.

**Figure 10:**
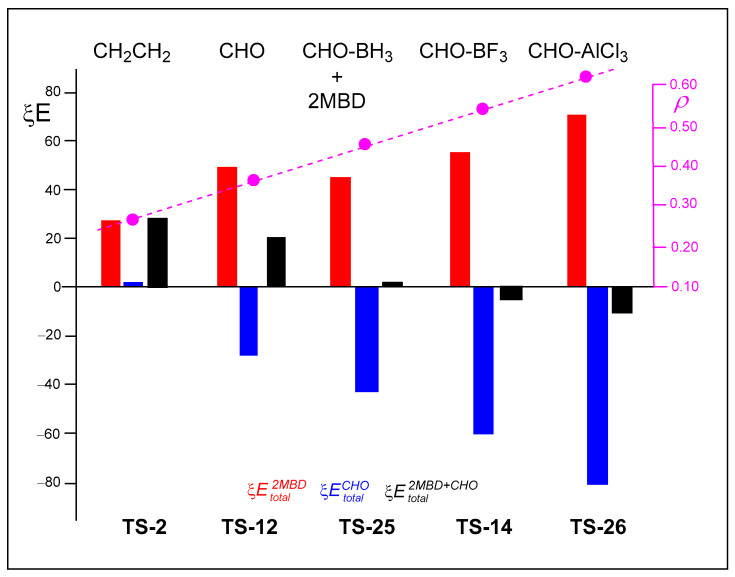
Graphical representation of the total energies, ξ$E_{total}^{CHO}$ ξ$E_{total}^{2MBD}$, and ξ$E_{total}^{2MBD+CHO}$ for the TSs given in Table 8. $E_{total}^{2MBD+CHO}$ values correspond to the RIAE activation energies of these AE reactions. ξ$E_{total}^{X}$ energies for the 2MBD and CHO frameworks are shown in red and blue, respectively, while the black bar represents the $E_{total}^{2MBD+CHO}$ relative energies. Energies are reported in kcal·mol^−1^, and GEDT values at the TSs are shown in pink as the average number of electrons e.

Finally, Figure 10 shows a graphical representation of the ξ$E_{total}^{X}$ energies of the 2MBD and CHO frameworks at the TSs, shown in red and blue, respectively. The ξ$E_{total}^{2MBD+CHO}$ energies, shown in black, represent the RIAE relative energies of these AE reactions.

Along this series of AE reactions, the ξ$E_{total}^{CHO}$ energies associated with the electrophilic CHO framework become more negative, i.e., more favorable, while those associated with the nucleophilic 2MBD framework, ξ$E_{total}^{2MBD}$, become more positive, i.e., more unfavorable. With the inclusion of the LA catalyst, the stabilization of the CHO:LA framework outweighs the destabilization of the 2MBD one, consequently decreasing the ξ$E_{total}^{2MBD+CHO}$ energies associated with these LA-catalyzed AE reactions, and even rendering these RIAE relative energies negative with the use of strongly acidic LAs (see Figure 10). These behaviors result from the GEDT that takes place at the TSs (see Figure 9); while the GEDT destabilizes the nucleophilic 2MBD framework due to the electron density loss, it stabilizes the electrophilic CHO:LA through electron density gain [27,45]. This behavior aligns with Parr’s electrophilicity ω index definition [33,41].

Many studies of P-DA reactions, including LA-catalyzed DA reactions, have shown that increasing the polar character of the reaction induces greater asynchronicity at the TSs, thereby increasing the distance between the two non-interacting centers [15]. This behavior, caused by an increase in the nucleophilic/electrophilic two-center interactions at the polar TSs, could contribute to the apparent reduction in proposed ‘Pauli repulsions’ within the framework of the ASM [46].

## 3. Conclusions

This MEDT study offers a thorough analysis of the LA-catalyzed AE reactions of 2MBD **15** with CHO **12**. The results emphasize several key findings: (i) *role of GEDT in reducing activation barriers.* The GEDT, primarily localized at the carbonyl C1 atom, acts as the main factor responsible for lowering the activation barriers. Increasing the acidic strength of the LA enhances the electrophilic nature of the CHO:LA complex, which in turn boosts GEDT and decreases the activation energy; (ii) *electronic stabilization shown by RIAE analysis.* The RIAE study indicates that although the nucleophilic 2MBD framework becomes more destabilized, the stabilization of the electrophilic CHO:LA framework dominates this effect. Notably, for BF_3_ and AlCl_3_, negative RIAE activation energies suggest that the TSs are electronically more stabilized than the separate reactants; (iii) *comparison with non-catalyzed processes.* The uncatalyzed AE reaction has a relatively high activation barrier because of the moderate electrophilicity of CHO. However, when LAs coordinate, the reaction speeds up considerably, with relative rate constants (k_r_) reaching up to 10^21^ times greater than the uncatalyzed process; and finally, (iv) *complementary use of ELF, AIM, and RIAE.* The combined application of ELF, AIM, and RIAE topological analyses provides a consistent mechanistic picture, confirming that LA catalysis changes the character of the TSs by stabilizing the electrophilic framework and promoting earlier C-C single bond formation.

In summary, this MEDT study demonstrates a direct connection between the acidity of the LA, the extent of GEDT, and the stabilization of the TS. These results reinforce the ability of MEDT to predict polar AE reactions and offer new insights into the design of LA-catalyzed procedures in organic synthesis.

## 4. Computational Details

The *ω*B97X-D [47] functional, which incorporates long-range exchange and semi-classical London dispersion corrections, has been shown to provide a more accurate description of kinetics, GEDT, and covalent processes compared to the B3LYP [48,49] functional. For the AIMAll [50] calculations, some stationary points were also computed using the M06-2X [51] functional. The standard 6-311G(d,p) basis set [52], which incorporates d-type polarization for second-row elements and p-type polarization functions for hydrogens, was employed throughout this MEDT study. The TSs were identified by the presence of a single imaginary frequency. Optimizations were performed using the Berny algorithm [53,54]. Intrinsic reaction coordinate [55] (IRC) paths were traced to map the energy profiles linking each TS to the two corresponding minima on the PES.

Solvent effects of dioxane were incorporated into the thermodynamic calculations by complete optimization of gas-phase stationary points at the same computational level using the polarizable continuum model [56,57] (PCM) within the framework of the self-consistent reaction field [58,59,60] (SCRF). Enthalpies, entropies, and Gibbs free energies at the *ω*B97X-D/6-311G(d,p) level in dioxane were computed using standard statistical thermodynamics at 25 °C and 1 atm [52]. These values were obtained from PCM frequency calculations performed on the solvent-optimized structures.

The GEDT [16] values were calculated using the equation GEDT(f) = Σq_f_, where q represents the natural charges [37,38] of the atoms in one of the two frameworks (f) at the TS geometries. DFT-based reactivity indices [34,35] were determined at the B3LYP/6-31G(d) level, following the equations outlined in [35], as the original electrophilicity and nucleophilicity scales were established using this computational method.

All calculations were carried out using the Gaussian 16 suite of programs [61]. ELF [17] analyses of the *ω*B97X-D/6-311G(d,p) monodeterminantal wavefunctions were performed with the TopMod [62] package employing a cubic grid with a step size of 0.1 Bohr. Molecular geometries and ELF basin attractors were visualized using the GaussView program [63]. The IQA analysis was performed using the AIMAll package [50] and the corresponding M06-2X/6-311G(d,p) monodeterminantal pseudo-wavefunctions. Note that the AIMAll package only allows the use of the B3LYP and the M06-2X functionals.

## Figures and Tables

**Table 1:** Populations, expressed as the average number of electrons, e, of the most relevant valence basins of 2MBD **15**, CHO **12**, and CHO:LA complexes **14**, **25**, and **26**.

	**a**	**a’**	**b**	**c**	**c’**
	V(C3,C4)	V’(C3,C4)	V(C4,C7)	V(C7,C8)	V’(C7,C8)
2MBD **15**	1.71	1.71	2.22	1.70	1.70
	**d**	**d’**	**e**	**e’**	
	V(C1,O2)	V’(C1,O2)	V(O2)	V’(O2)	
CHO **12**	1.23	1.23	2.54	2.54	
CHO:BH_3_ **25**	1.27	1.23	2.13	2.83	
CHO:BF_3_ **14**	1.21	1.25	2.26	2.79	
CHO:AlCl_3_ **26**	2.38		2.38	2.43	

**Table 2:** Electronic chemical potential μ, chemical hardness η, electrophilicity ω, and nucleophilicity *N* indices, calculated at the B3LYP/6-31G(d) level for 2MBD **15**, isobutene **10**, 1-butene **18**, propene **1,** ethylene **2**, CHO **12**, MeCHO **19**, CHO:LA complexes **14**, **25**, **26**, and MeCHO:AlCl_3_ **21**, expressed in eV.

	μ	η	ω	*N*
CHO:AlCl_3_ **26**	−6.09	4.27	4.35	0.89
MeCHO:AlCl_3_ **21**	−5.78	4.61	3.62	1.04
CHO:BH_3_ **25**	−5.29	4.87	2.87	1.40
CHO:BF_3_ **14**	−5.96	6.53	2.73	−0.11
CHO **12**	−4.23	6.16	1.45	1.81
MeCHO **19**	−3.77	6.35	1.12	2.18
2MBD **15**	−3.34	5.63	0.99	2.97
Ethylene **2**	−3.37	7.77	0.73	1.86
1-Butene **18**	−3.05	7.50	0.62	2.32
Propene **1**	−3.01	7.57	0.60	2.32
isobutene **10**	−2.83	7.37	0.55	2.60

**Table 3:** QTAIM parameters, in atomic units a.u., of the (3,−1) CPs at **TS-12**.

	CP-1	CP-2	CP-3
Density ρ	0.2765	0.1880	0.1031
Laplacian ∇^2^*ρ*	−0.9329	−0.4242	0.1032

**Table 4:** *ω*B97X-D/6-311G(d,p) relative energies, in kcal·mol^−1^, of the stationary points involved in the LA-catalyzed AE reactions of 2MBD **15** with CHO **12**.

	25		14	26
	BH_3_		BF_3_	AlCl_3_
**MC-25**	−10.3	**MC-X**	−11.7	−13.7
**TS-25**	3.3	**TS1-X**	−4.3	−10.3
**P-25**	−25.4	**IN-X**	−4.3	−10.8
		**TS2-X**	−3.2	−9.5
		**P-X**	−28.1	−30.4

**Table 5:** Main geometrical parameters of the TSs and intermediates involved in the BH_3_, BF_3_ and AlCl_3_ LA-catalyzed AE reactions of 2MBD **15** with CHO **12**. The GEDT at **TS-25**, **TS1-X** and **IN-X** are also included. Distances are given in Angstroms, while the GEDT is given as the average number of electrons, e.

		C1-C3	C5-H6	O2-H6	GEDT
BH_3_	**TS-25**	1.656	1.190	1.579	0.41
BF_3_	**TS1-14**	1.827	1.109	2.101	0.48
	**IN-14**	1.741	1.116	2.008	0.56
	**TS2-14**	1.629	1.203	1.565	
AlCl_3_	**TS1-26**	1.692	1.116	2.041	0.63
	**IN-26**	1.678	1.120	1.997	0.64
	**TS2-26**	1.609	1.225	1.516	

**Table 6:** *ω*B97X-D/6-311G(d,p) relative electronic energies, ΔE in kcal·mol^−1^, enthalpies, ΔH in kcal·mol^−1^, entropies, ΔH in cal·mol^−1^K^−1^, and Gibbs free energies, ΔG in kcal·mol^−1^, computed at 25 °C in dioxane for the stationary points involved in the AE reactions of 2MBD **15** with CHO **12**, and with the CHO:LA complexes **14**, **25** and **26**.

	ΔE	ΔH	ΔS	ΔG
**MC-12**	−6.7	−4.6	−26.6	3.4
**TS-12**	21.0	21.3	−42.7	34.0
**P-12**	−19.2	−14.8	−38.8	−3.3
**MC-25**	−9.2	−7.1	−29.9	1.8
**TS-25**	1.6	2.2	−45.5	15.7
**P-25**	−24.2	−19.9	−39.8	−8.0
**MC-14**	−10.9	−8.6	−34.9	1.8
**TS1-14**	−6.6	−4.4	−43.1	8.4
**IN-14**	−8.3	−5.5	−44.9	7.9
**TS2-14**	−6.2	−5.3	−48.6	9.2
**P-14**	−27.2	−23.0	−42.0	−10.5
**MC-26**	−12.1	−9.8	−36.3	1.0
**TS1-26**	−10.1	−8.0	−42.0	4.5
**IN-26**	−14.3	−11.5	−42.0	1.0
**TS2-26**	−11.1	−11.1	−52.3	4.5
**P-26**	−28.7	−24.7	−40.9	−12.5

**Table 7:** RRRCs, k_r_, for LA-catalyzed AE reactions of 2MBD **15** with the CHO:LA complexes **14**, **25** and **26**. The AE reaction of 2MBD **1** with CHO **2** has been taken as a reference.

**TS-12**	1.0
**TS-25**	2.5 × 10^13^
**TS2-24**	1.5 × 10^18^
**TS1-16**	4.5 × 10^21^

**Table 8:** M06-2X/6-311G(d,p) gas-phase ξ$E_{intra}^{X}$ intra-atomic, ξ$E_{inter}^{X}$ interatomic and ξ$E_{total}^{X}$ total energies, in kcal·mol^−1^, of the 2MBD and CHO frameworks, calculated at the M06-2X/6-311G(d,p) level of theory, at the TSs relative to their reagents in the GS. The sum of the ξ$E_{total}^{X}$ values of both frameworks, denoted as ξ$E_{total}^{2MBD+CHO}$, represents the RIAE relative energy.

Reagents	TS	ƒ(X)	$\xi E_{intra}^{X}$	$\xi E_{inter}^{X}$	$\xi E_{total}^{X}$	$\xi E_{total}^{2MBD+CHO}$
Ethylene **2**	**TS-2**	2MBD	44.24	−16.94	27.30	29.10
		Et	31.00	−29.12	1.88	
CHO **12**	**TS-12**	2MBD	77.56	−28.68	48.88	20.70
		CHO	−109.50	81.33	−28.18	
CHO:BH_3_ **25**	**TS-25**	2MBD	49.13	−4.19	44.94	1.54
		CHO:LA	−76.27	32.87	−43.40	
CHO:BF_3_ **14**	**TS1-14**	2MBD	53.85	0.68	54.53	−5.02
		CHO:LA	−83.58	24.02	−59.56	
CHO:AlCl_3_ **26**	**TS1-26**	2MBD	60.52	10.52	71.04	−10.05
		CHO:LA	−121.99	40.90	−81.09	

## Data Availability

The data underlying this study are available in the published article and its Supplementary Materials.

## Supplementary Materials

The following supporting information can be downloaded at: https://www.mdpi.com/article/10.3390/molecules30214289/s1. Theoretical background of the Relative Interacting Atomic Energy (RIAE) Analysis. Figure with the *ω*B97X-D/6-311G(d,p) ELF basin attractor positions and populations of the more relevant valence basins of **P-26**. Table with the *ω*B97X-D/6-311G(d,p) electronic chemical potential μ, chemical hardness η, electrophilicity ω and nucleophilicity *N* indices for 2MBD **15**, CHO **12**, ethylene **2**, and CHO:LA complexes **14**, **25**, and **26**. Table with the *ω*B97X-D/6-311G(d,p) total energies of the stationary points involved in the AE reactions of 2MBD **15** with ethylene **2**, CHO **12**, and the CHO:LA complexes **14**, **25** and **26**. Table with the *ω*B97X-D/6-311G(d,p) electronic energies, enthalpies, entropies, and Gibbs free energies computed a 25 ºC in dioxane, of the stationary points involved in the AE reactions of 2MBD **15** with CHO **12**, and the CHO:LA complexes **14**, **25** and **26**. Table with the main geometrical parameters of the TSs and intermediates in dioxane involved in the BH_3_, BF_3_ and AlCl_3_ LA-catalyzed AE reactions of 2MBD **15** with CHO **12**. Distances are given in Angstroms. *ω*B97X-D/6-311G(d,p) computed total energies, single imaginary frequency, and Cartesian coordinates of the stationary points involved in the AE reactions of 2MBD **15** with CHO **12**, and those involved in the AE reactions of 2MBD **15** with the CHO:LA complexes **14**, **25,** and **26**. References [64,65,66] are cited in the Supplementary Materials.
