# Supplementary material for: Unveiling the Role of the Lewis Acids in the Acceleration of Alder-Ene Reactions: A Molecular Electron Density Theory Study
Source: Molecules. 2025 Nov 4;30(21):4289. doi: 10.3390/molecules30214289 (PMC12609831; doi:10.3390/molecules30214289)
Supplement: Supplementary file 1 [file molecules-30-04289-s001.zip › molecules-3906913-supplementary.pdf]

Supplementary Material

Unveiling the Role of the Lewis Acids in the Acceleration of Alder-Ene Reactions: A Molecular Electron Density Theory Study

Luis R. Domingo [1,*] and Patricia Pérez [2,*]

[1] Independent Researcher, Avd. Tirso de Molina 20, 46015 Valencia, Spain
[2] Facultad de Ciencias, Departamento de Ciencias Biológicas y Químicas, Universidad San Sebastián,
Campus Ciudad Universitaria, Av. del Condor 720, Ciudad Empresarial, Huechuraba,
Santiago 8580704, Chile
* Correspondence: luisrdomingo@gmail.com (L.R.D.); patricia.perez@uss.cl (P.P.)

Index

S2 Theoretical background of the Relative Interacting Atomic Energy (RIAE) Analysis.

S4 Figure with the *ω*B97X-D/6-311G(d,p) ELF basin attractor positions and populations of the most relevant valence basins of *s-trans* butadiene.

S4 Figure with the *ω*B97X-D/6-311G(d,p) ELF basin attractor positions and populations of the most relevant valence basins of **P-14**.

S5 Table with the *ω*B97X-D/6-311G(d,p) electronic chemical potential μ, chemical hardness η, electrophilicity ω and nucleophilicity *N* indices for 2MBD **15**, CHO **12**, ethylene **2**, and CHO:LA complexes **14**, **25**, and **26**.

S6 Table with the *ω*B97X-D/6-311G(d,p) total energies of the stationary points involved in the AE reactions of 2MBD **15** with ethylene **2**, CHO **12**, and the CHO:LA complexes **14**, **25** and **26**.

S7 Table with the *ω*B97X-D/6-311G(d,p) electronic energies, enthalpies, entropies, and Gibbs free energies computed a 25 ºC in dioxane, of the stationary points involved in the AE reactions of 2MBD **15** with CHO **12**, and the CHO:LA complexes **14**, **25** and **26.**

S8 Table with the main geometrical parameters of the TSs and intermediates in dioxane involved in the BH_3, BF_3 and $AlCl_3$ LA-catalyzed AE reactions of 2MBD **15** with CHO **12**. Distances are given in Angstroms.

S9 *ω*B97X-D/6-311G(d,p) computed total energies, single imaginary frequency, and Cartesian coordinates of the stationary points involved in the AE reactions of 2MBD **15** with CHO **12**.

S11 *ω*B97X-D/6-311G(d,p) computed total energies, single imaginary frequency, and Cartesian coordinates of the stationary points involved in the AE reactions of 2MBD **15** with the CHO:LA complexes **14**, **25,** and **26**.

Theoretical background of the Relative Interacting Atomic Energy (RIAE) Analysis.

The Interacting Quantum Atoms [1] (IQA), based on the Quantum Theory of Atoms in Molecules [2] (QTAIM), divides the E_{total}^{IQA} total energy into two main energy contributions: the E_{intra}^{A} intra-atomic energies and the E_{inter}^{AB} interatomic energies (see Equation S1). The E_{inter}^{AB} energies are, in turn, divided into four additional electrostatic terms: the V_{ne}^{AB} and V_{en}^{AB} nuclei-electron interactions, the V_{ee}^{AB} electron-electron interactions, and the V_{nn}^{AB} nuclei-nuclei interactions (see Equation S3).

$$E_{total}^{IQA} = \sum E_{intra}^{A} + \sum E_{inter}^{AB} \quad \text{(S1)}$$

$$E_{intra}^{A} = T(A) + V_{ne}^{A} + V_{ee}^{A} \quad \text{(S2)}$$

$$E_{inter}^{AB} = ½V_{ne}^{AB} + ½V_{en}^{AB} + ½V_{ee}^{AB} + V_{nn}^{AB} \quad \text{(S3)}$$

Thanks to the additivity of the topological atoms [3], an IQF approach has been recently introduced [4], which allows the grouping of the IQA energy in terms of convenient fragments of the system. This enables a more chemically meaningful energy analysis of the interactions that take place between the atoms forming groups. In this sense, in the so-called Relative Interacting Atomic Energy [5] (RIAE) analysis of the AE reactions, the atoms belonging to the TSs are regrouped in the two interacting frameworks f(X) related to the propene and ethylene or carbonyl reagents [6,7].

By default, the sum of all IQA atomic energies belonging to the considered framework f(X) (where X represents either the propene or ethylene/carbonyl frameworks) at the TSs, and those of the separated reagents at the ground states (GSs), is computed. The RIAEs, i.e., the relative ξE_{total}^{X} total, ξE_{intra}^{X} intra-atomic, and ξE_{inter}^{X}interatomic energies, are obtained using Equations S4-S6. The symbol ξ denotes the IQA energy differences between the GS and the TS states of the two-interacting frameworks f(X); i.e. f(2MBD) and f(CHO) in these AE reactions.

$$\xi E_{total}^{X} = \xi E_{intra}^{X} + \xi E_{inter}^{X} \quad \text{(S4)}$$

$$\xi E_{intra}^{X} = \sum E_{intra}^{X(TS)} - \sum E_{intra}^{X(GS)} \quad \text{(S5)}$$

$$\xi E_{inter}^{X} = \sum E_{inter}^{X(TS)} - \sum E_{inter}^{X(GS)} \quad \text{(S6)}$$

The herein proposed RIAE analysis provides a measure of how much the two interacting frameworks f(X) are destabilized (resulting in positive relative energies) or stabilized (resulting in negative relative energies) when going from their GS to the TS. The sum of the ξE_{total}^{X} energies of the two interacting frameworks, ξE_{total}^{2MBD+} , provides the RIAE relative energy of AE reactions obtained through the present energy decomposition analysis [5-7].

References

1. Blanco, M. A.; Martín Pendás, A.; Francisco, E. Interacting Quantum Atoms: A Correlated Energy Decomposition Scheme Based on the Quantum Theory of Atoms in Molecules, *J. Chem. Theory Comput.* **2005**, *1*, 1096–1109.
2. (a) Bader, R.F.W.; Tang, Y.H.; Tal, Y.; Biegler-König, F.W. Properties of atoms and bonds in hydrocarbon molecules. *J. Am. Chem. Soc.* **1982**, *104*, 946–952; (b) Bader, R.F.W. *Atoms in Molecules: A Quantum Theory*, Oxford University Press, Oxford, New York, 1994.
3. Martín Pendás, A.; Blanco, M.A.; Francisco, E. Chemical Fragments in Realpace: Definitions, Properties and Energetic Decompositions. *J. Comput. Chem.* 2007, **28**, 161–184.
4. Triestram, L.; Falcioni, F.; Popelier, P. L. A. Interacting Quantum Atoms and Multipolar Electrostatic Study of XH···π Interactions, *ACS Omega* **2023,** *8*, 34844–34851.
5. Domingo, L.R.: Ríos-Gutiérrez, M.; Pérez, P.; Understanding the Electronic Effects of Lewis Acid Catalysts in Accelerating Polar Diels-Alder Reactions. *J. Org. Chem.* **2024**. 89, 12349-12359.
6. Domingo, L.R.; Pérez, P.; Ríos-Gutiérrez, M.; Aurell, M.J. A Molecular Electron Density Theory Study of Hydrogen Bond Catalysed Polar Diels–Alder Reactions of α,β-unsaturated Carbonyl Compounds, *Tetrahedron Chem.* **2024**, *10*,100064.
7. Domingo, L.R.; Ríos-Gutiérrez, Revealing the Decisive Role of Global Electron Density Transfer in the Reaction Rate of Polar Organic Reactions within Molecular Electron Density Theory, *Molecules* **2024**, *29*, 1.

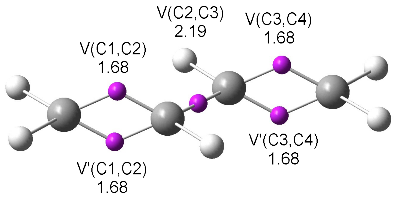

Figure S1. *ω*B97X-D/6-311G(d,p) ELF basin attractor positions and populations of the most relevant valence basins of *s-trans* butadiene.

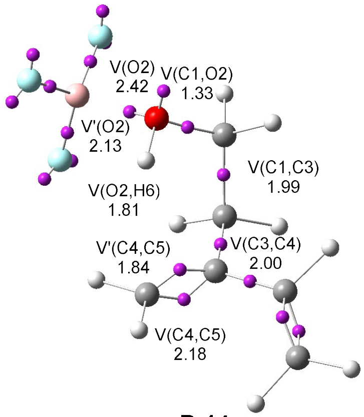

P-14

Figure S2. *ω*B97X-D/6-311G(d,p) ELF basin attractor positions and populations of the most relevant valence basins of **P-14**.

Table S1. Electronic chemical potential μ, chemical hardness η, electrophilicity ω and nucleophilicity *N* indices, calculated at the *ω*B97X-D/6-311G(d,p) for 2MBD **15**, CHO **12**, ethylene **2**, and CHO:LA complexes **14**, **25** and **26**, expressed in eV.

	μ	η	ω	*N*
CHO:$AlCl_3$ **26**	-6.22	8.70	2.22	0.82
CHO:BH_3 **25**	-6.14	10.95	1.72	-0.23
CHO:BF_3 **14**	-5.47	9.33	1.60	1.26
CHO **12**	-4.33	10.55	0.89	1.78
2MBD **15**	-3.65	9.59	0.69	2.94
Ethylene **2**	-3.75	11.96	0.59	1.66

Table S2. *ω*B97X-D/6-311G(d,p) total energies, in a.u., of the stationary points involved in the AE reactions of 2MBD **15** with ethylene **2**, CHO **12**, and the CHO:LA complexes **14**, **25** and **26**.

**2**	-78.579526	**12**	-114.494221		
**15**	-195.286367				
**MC-2**	-273.873998	**MC-12**	-309.792164		
**TS-2**	-273.819020	**TS-12**	-309.746671		
**P-2**	-273.911099	**P-12**	-309.811857		
**25**	-141.126112	**14**	-439.068161	**26**	-1737.798667
**MC-25**	-336.428841	**MC-14**	-634.373115	**MC-26**	-1933.106809
**TS-25**	-336.407165	**TS1-14**	-634.361328	**TS1-26**	-1933.100420
**P-25**	-336.452941	**IN-14**	-634.361320	**IN-26**	-1933.102263
		**TS2-14**	-634.359658	**TS2-26**	-1933.100139
		**P-14**	-634.399357	**P-26**	-1933.133400

Table S3. *ω*B97X-D/6-311G(d,p) electronic energies, E in a.u., enthalpies, H in a.u., entropies, S in cal·mol^{-1}K^{-1}, and Gibbs free energies, G in a.u., computed a 25 ºC in dioxane, of the stationary points involved in the AE reactions of 2MBD **15** with CHO **12**, and the CHO:LA complexes **14**, **25** and **26**.

	E	H	S	G
**15**	-195.287344	-195.167676	72.5	-195.202118
**12**	-114.496401	-114.465731	53.6	-114.491192
**MC-12**	-309.794371	-309.640693	99.5	-309.687967
**TS-12**	-309.750337	-309.599471	83.4	-309.639109
**P-12**	-309.814268	-309.657059	87.3	-309.698523
**25**	-141.131841	-141.066257	64.5	-141.096919
**MC-25**	-336.433815	-336.245319	107.2	-336.296240
**TS-25**	-336.416693	-336.230463	91.5	-336.273945
**P-25**	-336.457726	-336.265638	97.2	-336.311822
**14**	-439.074080	-439.024177	79.5	-439.061955
**MC-14**	-634.378792	-634.205545	117.1	-634.261187
**TS1-14**	-634.371923	-634.198904	108.9	-634.250642
**IN-14**	-634.374598	-634.200628	107.1	-634.251504
**TS2-14**	-634.371304	-634.200335	103.4	-634.249456
**P-14**	-634.404810	-634.228537	110.0	-634.280820
**26**	-1737.806990	-1737.762133	95.1	-1737.807333
**MC-26**	-1933.113609	-1932.945414	131.2	-1933.007799
**TS1-26**	-1933.110369	-1932.942599	125.7	-1933.002308
**IN-26**	-1933.117072	-1932.948112	125.7	-1933.007816
**TS2-26**	-1933.111976	-1932.947506	115.3	-1933.002282
**P-26**	-1933.140014	-1932.969091	126.7	-1933.029312

Table S4. Main geometrical parameters of the TSs and intermediates in dioxane involved in the BH_3, BF_3 and $AlCl_3$ LA-catalyzed AE reactions of 2MBD **15** with CHO **12**. Distances are given in Angstroms.

		C1–C3	C6–H7	O2–H7
SC	**TS-12**	1.849	1.229	1.443
BH_3	**TS-25**	1.628	1.207	1.542
BF_3	**TS1-14**	1.963	1.101	2.354
	**IN-14**	1.660	1.118	2.010
	**TS2-14**	1.598	1.240	1.482
$AlCl_3$	**TS1-26**	2.041	1.096	2.746
	**IN-26**	1.635	1.114	2.080
	**TS2-26**	1.586	1.264	1.439

ωB97X-D/6-311G(d,p) computed total energies, single imaginary frequency, and Cartesian coordinates of the stationary points involved in the AE reactions of 2MBD **15** with CHO **12**.

15
E(RwB97XD) = -195.286367 A.U.

C	0.63361200	1.69399400	0.00000000
H	0.08631700	2.63096100	0.00000000
H	1.71664100	1.75542200	0.00000000
C	0.00341100	0.51584600	0.00000000
C	-1.50736900	0.45178300	0.00000000
H	-1.91142100	0.95322800	0.88255500
H	-1.91142100	0.95322800	-0.88255500
H	-1.88791000	-0.56882000	0.00000000
C	0.80337300	-0.71805300	0.00000000
C	0.35591800	-1.97228000	0.00000000
H	1.87956100	-0.55749900	0.00000000
H	1.04916100	-2.80521400	0.00000000
H	-0.69890000	-2.22221800	0.00000000

12
E(RwB97XD) = -114.494221 A.U.

C	-0.00005900	-0.52042000	-0.00000100
O	0.00001800	0.67587100	0.00000100
H	0.00001200	-1.10986100	0.93885500
H	0.00001200	-1.10986300	-0.93885600

MC-12
E(RwB97XD) = -309.792164 A.U.

O	-2.57400400	-0.07825700	0.72770200
C	-2.37265800	-0.41062000	-0.40665400
C	1.81565200	-1.31910300	-0.78723700
H	-0.27893100	-0.43704100	1.47658000
C	0.80024100	-0.57399900	1.35656500
H	2.02577600	-2.29552800	-0.36453400
H	2.11606300	-1.15135000	-1.81663700
C	1.22008400	-0.35993500	-0.07206200
H	1.06190400	-1.57806800	1.69330700
H	1.29353600	0.14886200	2.01369000
H	-3.15501800	-0.31837200	-1.18588700
H	-1.40084300	-0.82488500	-0.73526800
C	0.95591800	0.94815700	-0.69186000
C	0.29735500	1.95568400	-0.12122300
H	1.33718200	1.07262900	-1.70332200
H	0.14286600	2.89049800	-0.64752100

H	-0.11373800	1.88292100	0.87972000

TS-12
E(RwB97XD) = -309.746671 A.U.
Imaginary frequency -1032.1138 cm^{-1}

O	-1.95964200	0.62909900	-0.77930200
C	-1.78499900	-0.61078200	-0.51125500
C	-0.64946200	-0.83290400	0.96799600
H	-0.81133900	1.28362600	-0.27465600
C	0.14627200	1.38613600	0.50478900
H	-1.30584700	-0.43773300	1.73729300
H	-0.47792900	-1.90448200	1.03032000
C	0.41014700	-0.01279200	0.55555700
H	-0.34909200	1.80785300	1.37755000
H	0.91323100	2.03389700	0.08845300
H	-2.61755300	-1.13976400	-0.02440900
H	-1.26750600	-1.24129700	-1.25517800
C	1.53388900	-0.62905000	-0.15491300
C	2.65823800	-0.00494500	-0.50229400
H	1.43178500	-1.69024600	-0.36981500
H	3.45612000	-0.53450300	-1.00921500
H	2.82835500	1.04266700	-0.27950900

P-12
E(RwB97XD) = -309.811857 A.U.

C	-1.78274100	-0.78026800	-0.03850500
O	-2.32423800	0.30391200	-0.75518400
C	-0.72778900	-0.33536400	0.98342800
H	-1.19013000	0.38623900	1.66155700
H	-0.41519300	-1.20206400	1.57517700
C	0.46667500	0.29008200	0.30284000
C	0.55377400	1.61106500	0.12163200
H	-1.58797500	0.78824200	-1.13783200
H	-0.18363400	2.28350700	0.54722800
H	1.36549900	2.05349400	-0.44557000
H	-2.61771700	-1.25235200	0.48299700
H	-1.35240800	-1.53109600	-0.71873800
C	1.47868900	-0.64589500	-0.22681800
C	2.78728700	-0.41429800	-0.27236300
H	1.10417500	-1.61069900	-0.56556600
H	3.47525500	-1.14891900	-0.67445900
H	3.21183900	0.51080200	0.10455800

ωB97X-D/6-311G(d,p) computed total energies, single imaginary frequency, and Cartesian coordinates of the stationary points involved in the AE reactions of 2MBD **15** with the CHO:LA complexes **14**, **25**, and **26**.

25

E(RwB97XD) = -141.126112 A.U.

C	0.01118500	0.02460200	0.00381000
O	0.05068600	-0.09429800	1.20993700
H	0.93140900	0.17234800	-0.57413400
H	-0.95902800	-0.01756600	-0.50044700
B	1.47991200	-0.04372800	2.06254300
H	2.31406300	0.12463500	1.19824300
H	1.29704300	0.88791400	2.80381800
H	1.49038800	-1.12239900	2.59857100

MC-25

E(RwB97XD) = -336.428841 A.U.

C	-0.27268200	-1.05520600	-1.04291800
O	-0.96035800	-0.05058000	-1.08269200
C	1.16019900	-0.74477500	1.44513200
H	0.24429000	-0.38606900	1.90327500
H	1.47177000	-1.75854500	1.67890300
C	1.89135000	0.03637500	0.63746900
C	1.47978700	1.44802500	0.32868500
H	2.25268700	2.15166100	0.65129500
H	1.33583600	1.59128300	-0.74703600
H	0.54273400	1.69956500	0.82498700
H	-0.55117500	-1.89902900	-0.40416900
H	0.60983100	-1.11841100	-1.68487400
B	-2.23276400	0.15445200	-0.07019400
C	3.11897800	-0.49586300	0.02597600
C	3.89207400	0.15377900	-0.84267600
H	3.39128600	-1.50715500	0.32096300
H	4.78209300	-0.30835900	-1.25319400
H	3.67213600	1.16543500	-1.16650100
H	-1.84946000	1.05962900	0.63512700
H	-3.14099800	0.44896600	-0.80376000
H	-2.33958200	-0.91561200	0.49398400

TS-25

E(RwB97XD) = -336.407165 A.U.

Imaginary frequency -447.5031 cm^{-1}

C	0.16420000	1.31194300	-0.56216800
O	0.80449500	0.17598100	-0.89550200
C	-0.82739900	1.11030300	0.74897600

H	-0.12501500	0.83584600	1.53676700
H	-1.31851500	2.06379300	0.93885600
C	-1.71456800	0.04020300	0.37493500
C	-1.12252100	-1.24532000	0.23879200
H	-0.28149000	-0.91155400	-0.53459800
H	-0.44124100	-1.52830900	1.04482900
H	-1.73502900	-2.05657800	-0.14306100
H	0.84814300	2.08692700	-0.20228800
H	-0.44948700	1.68435300	-1.38899900
B	2.02695500	-0.21908200	0.01070200
C	-3.04240100	0.36892600	-0.12049300
C	-4.00530800	-0.52941400	-0.34058500
H	-3.25405900	1.42482000	-0.26426900
H	-4.98693500	-0.22117900	-0.68031700
H	-3.85630800	-1.58933500	-0.16758500
H	2.45705900	-1.25195900	-0.45306500
H	2.80803500	0.71353500	-0.02218400
H	1.59758600	-0.38768800	1.15600600

P-25
E(RwB97XD) = -336.452941 A.U.

C	-0.09655400	-1.42405400	-0.18492900
O	-0.84861700	-0.37803300	-0.82625800
C	0.83403000	-0.86893500	0.89226900
H	0.23019500	-0.41909900	1.68124500
H	1.38942100	-1.70586200	1.32506800
C	1.77788600	0.15608800	0.30482600
C	1.45829400	1.45528700	0.30148400
H	-0.24657100	0.35563200	-1.01786000
H	0.56882700	1.81395500	0.81031000
H	2.08412200	2.18937300	-0.19412000
H	-0.84296700	-2.09495200	0.23676300
H	0.45741000	-1.94728800	-0.96875900
B	-2.17821100	0.22382400	-0.01626700
C	3.00238700	-0.36707100	-0.33247200
C	4.17679700	0.25572100	-0.35726500
H	2.92489000	-1.35886100	-0.77572800
H	5.03832900	-0.18823200	-0.84187700
H	4.31709400	1.22025600	0.12002700
H	-2.54821000	1.09691700	-0.76184100
H	-2.89956700	-0.73980500	0.06070000
H	-1.75235400	0.62107700	1.04712700

14
E(RwB97XD) = -439.068161 A.U.

C	-0.04113300	0.02397100	-0.03808600

O	-0.04746600	-0.09958400	1.16467400
H	0.89786200	0.16990700	-0.58764300
H	-0.98812200	-0.01035900	-0.59174500
F	1.53236200	-1.26382800	2.68761800
F	2.44196300	0.14495500	1.12283000
F	1.31246600	1.01402600	2.91981900
B	1.58042100	-0.04281200	2.14768800

MC-14
E(RwB97XD) = -634.373115 A.U.

C	-0.23723000	-1.03881100	-1.00052500
O	-0.94846700	-0.04962200	-1.08080100
C	1.14046300	-0.71449500	1.41817600
H	0.23051700	-0.33739600	1.87175800
H	1.44013600	-1.72894000	1.66456200
C	1.88921200	0.05318000	0.61116100
C	1.49794500	1.46898000	0.29533500
H	2.26294500	2.16427900	0.65271600
H	1.39971800	1.61894400	-0.78467400
H	0.54383100	1.72136600	0.75532900
H	-0.52393900	-1.88624700	-0.37268200
H	0.66477500	-1.09400200	-1.61559900
F	-2.49391600	-1.15459700	0.42605400
F	-1.79838400	0.97769800	0.90123300
F	-3.24800500	0.60469900	-0.85349800
B	-2.29989600	0.11902900	-0.03010600
C	3.11595700	-0.49853200	0.01529600
C	3.90916600	0.13942300	-0.84376300
H	3.37032400	-1.51299800	0.31531300
H	4.79813500	-0.33542900	-1.24181500
H	3.70822400	1.15405400	-1.17042500

TS1-14
E(RwB97XD) = -634.361328 A.U.
Imaginary frequency -107.5024 cm^{-1}

C	0.00000000	0.00000000	0.00000000
O	0.00000000	0.00000000	1.30212700
C	1.68653100	0.00000000	-0.70216200
H	2.07571600	-0.86824600	-0.18018100
H	1.50540100	-0.12552400	-1.76673800
C	2.11597600	1.25774000	-0.27501800
C	2.53556500	1.41709900	1.12547800
H	1.60143700	1.29721900	1.71092400
H	3.15777100	0.58588200	1.45404900
H	2.99292700	2.37546500	1.35957500
H	-0.27457300	-0.94470400	-0.47730200

H	-0.47187300	0.87760200	-0.44551300
F	-0.23935400	-2.35444300	1.32346900
F	1.77609100	-1.43735100	1.92231500
F	-0.04614300	-1.18271300	3.31244100
B	0.37417100	-1.32366600	2.02160400
C	1.93593300	2.39085800	-1.17126700
C	2.23675400	3.65828100	-0.87811600
H	1.53066000	2.15835800	-2.15221200
H	2.07448500	4.44621200	-1.60410300
H	2.65548200	3.95460100	0.07629500

IN-14
E(RwB97XD) = -634.361320 A.U.

C	0.00000000	0.00000000	0.00000000
O	0.00000000	0.00000000	1.32022400
C	1.60303300	0.00000000	-0.67857800
H	2.01731200	-0.88319700	-0.20040400
H	1.44635800	-0.08955100	-1.75164300
C	2.11280600	1.23959800	-0.23530100
C	2.52457900	1.35056700	1.16193100
H	1.56788200	1.19108900	1.71428400
H	3.12760400	0.49990900	1.47850000
H	2.96602500	2.30141100	1.44898100
H	-0.33696300	-0.93642800	-0.45520000
H	-0.50155300	0.87353000	-0.42374500
F	-0.33033400	-2.34039900	1.37518500
F	1.74877500	-1.50345500	1.86796800
F	0.00785600	-1.15696200	3.33966300
B	0.35240000	-1.32168600	2.02666100
C	1.99710000	2.38787000	-1.11689500
C	2.45944000	3.61093800	-0.84107400
H	1.52041100	2.20578900	-2.07587200
H	2.35209900	4.41673900	-1.55763100
H	2.96741400	3.84783500	0.08627400

TS2-14
E(RwB97XD) = -634.359658 A.U.
Imaginary frequency -458.2512 cm^{-1}

C	0.15008200	1.32668400	-0.48085300
O	0.76502700	0.16994300	-0.85800400
C	-0.84503500	1.11932300	0.79172400
H	-0.18565800	0.85353600	1.61499600
H	-1.36142700	2.06360900	0.96183100
C	-1.72216100	0.03287200	0.39600200
C	-1.14228800	-1.25590800	0.32526100
H	-0.29489500	-0.90792100	-0.45477800

H	-0.46246900	-1.51435000	1.13664800
H	-1.73832100	-2.07410600	-0.06673100
H	0.86871500	2.06862200	-0.12496800
H	-0.43478400	1.73377300	-1.31054600
F	2.92661600	0.83018700	-0.16586100
F	1.64115100	-0.41942000	1.27046600
F	2.46734000	-1.39330400	-0.64593000
B	2.03877700	-0.22309400	-0.07585500
C	-3.01338800	0.36043300	-0.18747200
C	-3.98473400	-0.53345100	-0.38809400
H	-3.19417900	1.40903100	-0.40572600
H	-4.94532100	-0.23163000	-0.78843900
H	-3.86497600	-1.58113400	-0.13565200

P-14
E(RwB97XD) = -634.399357 A.U.

C	-0.09400100	-1.42220000	-0.16973400
O	-0.83531100	-0.35531500	-0.80354800
C	0.85056200	-0.88251000	0.90200500
H	0.25809300	-0.44131900	1.70333000
H	1.41009100	-1.72713100	1.31385300
C	1.78942500	0.14849800	0.31535400
C	1.46868000	1.44758800	0.33182200
H	-0.22933000	0.38005600	-0.98748700
H	0.58505300	1.79706400	0.85595400
H	2.09084900	2.18813500	-0.15876900
H	-0.84919600	-2.08464200	0.24723300
H	0.44262700	-1.94469900	-0.96449500
F	-2.96414900	-0.88826200	0.12260800
F	-1.75442300	0.78545900	1.12616400
F	-2.65920900	1.14092500	-0.95215700
B	-2.22275600	0.23672400	-0.04246900
C	3.00656500	-0.36846900	-0.34049100
C	4.17981100	0.25603200	-0.37420200
H	2.92523600	-1.35691000	-0.79057800
H	5.03610100	-0.18305800	-0.87226700
H	4.32464200	1.21690400	0.10903700

26
E(RwB97XD) = -1737.798667 A.U.

C	-0.12357500	0.02593600	-0.13674700
O	-0.00561500	-0.08644900	1.06840300
H	0.75250400	0.17750500	-0.78037800
H	-1.12490700	-0.02553100	-0.57670900
Al	1.61080300	-0.04592200	2.20512600
Cl	1.19777700	1.61630500	3.41937000

Cl	1.54383700	-1.96417600	3.05592200
Cl	3.08540400	0.24866300	0.70665800

MC-26
E(RwB97XD) = -1933.106809 A.U.

C	-0.12238200	-0.93247700	-0.99661300
O	-0.95262800	-0.02754900	-1.00373500
C	1.18067700	-0.72827100	1.39784700
H	0.32709000	-0.31507900	1.92506200
H	1.41122000	-1.77451200	1.57470100
C	1.96120900	0.03430600	0.61263200
C	1.67444900	1.49336600	0.39994200
H	2.52958800	2.09825900	0.71388900
H	1.49402700	1.71186200	-0.65790200
H	0.79758300	1.80952700	0.96379100
H	-0.32203400	-1.88057900	-0.48807500
H	0.78704700	-0.81958200	-1.59145700
C	3.11953600	-0.57092800	-0.06350100
C	3.91789200	0.05534300	-0.92656600
H	3.31181600	-1.61514300	0.17336200
H	4.75220400	-0.45848100	-1.38901100
H	3.77750900	1.09770100	-1.19193000
Al	-2.59770300	0.12152400	-0.01621200
Cl	-2.12854000	1.66414300	1.35155800
Cl	-4.02107000	0.54057200	-1.50433300
Cl	-2.71398300	-1.80804300	0.87412300

TS1-26
E(RwB97XD) = -1933.100420 A.U.
Imaginary frequency -228.1052 cm^{-1}

C	0.08083600	0.04118300	-0.03580700
O	-0.00543800	0.01735500	1.24388700
C	1.95306800	-0.06623700	-0.62742400
H	2.26642300	-0.88404800	0.01369600
H	1.82319300	-0.30520900	-1.67868900
C	2.25528500	1.23343500	-0.27493700
C	2.59903400	1.54640700	1.13293300
H	1.66207900	1.50681900	1.71138900
H	3.23957100	0.77654100	1.56210200
H	3.05383200	2.52592100	1.26438300
H	-0.12997900	-0.88160400	-0.58525800
H	-0.25194600	0.95954600	-0.52229500
C	2.07199000	2.28227200	-1.27388900
C	2.16592300	3.59215500	-1.03870400
H	1.82757900	1.94243900	-2.27671700
H	2.00213300	4.30896000	-1.83455800

H	2.40532400	3.99582000	-0.06192200
Al	0.08722900	-1.45613300	2.35608500
Cl	-1.21883600	-1.06177600	3.96565500
Cl	2.15585300	-1.57650100	2.89812800
Cl	-0.46095000	-3.07301900	1.06710300

IN-26
E(RwB97XD) = -1933.102263 A.U.

C	0.10824800	-0.05615000	-0.16682300
O	0.07752800	-0.07671100	1.17020600
C	1.66544500	0.07736100	-0.77768000
H	2.17647800	-0.78971300	-0.36191500
H	1.55783300	0.03232500	-1.86069800
C	2.13094100	1.33385500	-0.28207500
C	2.54492500	1.39801100	1.11094600
H	1.59272100	1.13645400	1.63896300
H	3.20917300	0.57508000	1.38308500
H	2.91061400	2.35932900	1.46171000
H	-0.19838600	-0.99554000	-0.64521800
H	-0.46251100	0.78043800	-0.58440500
C	1.96278000	2.50404500	-1.11529400
C	2.41174200	3.72525000	-0.80052500
H	1.46879400	2.34793100	-2.06959000
H	2.27360800	4.55691000	-1.48140500
H	2.94110700	3.93366500	0.12177500
Al	0.25082200	-1.52528400	2.24130100
Cl	-1.08600300	-3.00291700	1.48426400
Cl	2.30812600	-2.15621600	1.97524200
Cl	-0.10405400	-0.82594200	4.21100800

TS2-26
E(RwB97XD) = -1933.100139 A.U.
Imaginary frequency -547.5685 cm^{-1}

C	0.21577200	-0.19503700	-0.19396000
O	0.13025300	-0.16338000	1.17726100
C	1.72526500	0.03400400	-0.70270300
H	2.28296000	-0.85455200	-0.40687100
H	1.69611000	0.13902400	-1.78702400
C	2.18215600	1.23577800	-0.01050800
C	2.45014500	1.10034100	1.36544900
H	1.30449000	0.71472600	1.56129500
H	2.96221400	0.18986900	1.67638000
H	2.67471500	1.98335600	1.95462100
H	-0.05702700	-1.17121700	-0.60668300
H	-0.42315700	0.57624000	-0.63484700
C	2.02578600	2.51941500	-0.67317900

C	2.60267100	3.64492800	-0.24417400
H	1.45731200	2.52310600	-1.59851900
H	2.49147800	4.57202500	-0.79385900
H	3.22230900	3.67267500	0.64529400
Al	0.08993200	-1.63397900	2.26228000
Cl	0.37955800	-0.80708400	4.19922200
Cl	-1.73370300	-2.65648100	1.90592100
Cl	1.80329400	-2.77219900	1.60852600

P-26

E(RwB97XD) = -1933.133400 A.U.

C	-0.01059900	-1.42519300	0.12037500
O	-0.75987200	-0.39417400	-0.57583400
C	1.00638100	-0.78369900	1.05953700
H	0.47198800	-0.26548400	1.85786100
H	1.59942400	-1.58279900	1.51249000
C	1.88803500	0.18301700	0.29992300
C	1.56789300	1.48105300	0.22861500
H	-0.14444900	0.30447800	-0.85894700
H	0.74541800	1.89124400	0.80705700
H	2.13750900	2.17157800	-0.38313000
H	-0.74557300	-2.01907300	0.66214700
H	0.45721900	-2.04686900	-0.64480200
C	3.04018400	-0.39995100	-0.41505800
C	4.20089400	0.21591300	-0.61718800
H	2.92350300	-1.42667400	-0.75841900
H	5.00981500	-0.27151100	-1.14812200
H	4.38453200	1.21817200	-0.24385000
Al	-2.53088800	0.19086700	-0.13273100
Cl	-2.40237100	0.50753000	1.96251500
Cl	-2.59469100	1.94815100	-1.30400300
Cl	-3.73345900	-1.44612700	-0.69150100
